# The Universal Form of Treatment Options (UFTO) as an Alternative to Do Not Attempt Cardiopulmonary Resuscitation (DNACPR) Orders: A Mixed Methods Evaluation of the Effects on Clinical Practice and Patient Care

**DOI:** 10.1371/journal.pone.0070977

**Published:** 2013-09-04

**Authors:** Zoë Fritz, Alexandra Malyon, Jude M. Frankau, Richard A. Parker, Simon Cohn, Clare M. Laroche, Chris R. Palmer, Jonathan P. Fuld

**Affiliations:** 1 Department of Acute Medicine, Cambridge University Hospitals NHS Foundation Trust, Cambridge, United Kingdom; 2 Centre for Applied Medical Statistics, University of Cambridge Institute of Public Health, Cambridge, United Kingdom; 3 Primary Care Unit, University of Cambridge Institute of Public Health, Cambridge, United Kingdom; 4 Department of Respiratory Medicine, West Suffolk Hospital, Bury St. Edmunds, Suffolk, United Kingdom; Cardiff University, United Kingdom

## Abstract

**Aims:**

To determine whether the introduction of the Universal Form of Treatment Options (the UFTO), as an alternative approach to Do Not Attempt Cardiopulmonary Resuscitation (DNACPR) orders, reduces harms in patients in whom a decision not to attempt cardiopulmonary resuscitation (CPR) was made, and to understand the mechanism for any observed change.

**Methods:**

A mixed-methods before-and-after study with contemporaneous case controls was conducted in an acute hospital. We examined DNACPR (103 patients with DNACPR orders in 530 admissions) and UFTO (118 decisions not to attempt resuscitation in 560 admissions) practice. The Global Trigger Tool was used to quantify harms. Qualitative interviews and observations were used to understand mechanisms and effects.

**Results:**

Rate of harms in patients for whom there was a documented decision not to attempt CPR was reduced: Rate difference per 1000 patient-days was 12.9 (95% CI: 2.6–23.2, p-value = 0.01). There was a difference in the proportion of harms contributing to patient death in the two periods (23/71 in the DNACPR period to 4/44 in the UFTO period (95% CI 7.8–36.1, p-value = 0.006). Significant differences were maintained after adjustment for known confounders. No significant change was seen on contemporaneous case control wards. Interviews with clinicians and observation of ward practice revealed the UFTO helped provide clarity of goals of care and reduced negative associations with resuscitation decisions.

**Conclusions:**

Introducing the UFTO was associated with a significant reduction in harmful events in patients in whom a decision not to attempt CPR had been made. Coupled with supportive qualitative evidence, this indicates the UFTO improved care for this vulnerable group.

**Trial Registration:**

Controlled-Trials.com ISRCTN85474986 UK Comprehensive Research Network Portfolio 7932

## Introduction

In the UK, there are on average 160,000 hospital deaths annually [1]. Of those, 80% die with a Do Not Attempt Cardiopulmonary Resuscitation (DNACPR) order in place [2], [3]. DNACPR orders exist to provide immediacy and clarity of instruction in the event of a cardiorespiratory arrest; they are written either at a patient’s request, or because a clinical decision has been made that a patient would be unlikely to survive attempted cardiopulmonary resuscitation.

The decision not to attempt CPR should not be conflated with decisions to initiate palliation or withhold other treatments; around 50% of patients with DNACPR orders are discharged from hospital [unpublished data].

All NHS Trusts use some kind of *proforma* to record a DNACPR decision. While practice varies, most follow the Resuscitation Council UK’s guidance, and a model DNACPR form was published in 2009. Documentation is placed at the front of the notes, with red demarcation common for rapid identification in an emergency [4].

Several problems exist with the current practice:

Firstly, there is evidence that DNACPR orders are often misinterpreted by doctors and nurses [5], leading to other treatments being inappropriately withheld [6]–[9] including echocardiograms for patients with heart failure [10] or admission of patients to ICU [11]. In-hospital mortality is higher in patients with DNACPR orders than for those with similar comorbidities and severity of illness without such orders in place [12]–[15].

Secondly, the recent UK National Confidential Enquiry into Patient Outcomes and Deaths (NCEPOD) report [16] highlighted the current ad hoc nature of resuscitation decision-making, revealing that many patients have resuscitation attempted on them inappropriately, because DNACPR orders are not completed when they should be. In many cases the reason given for not completing a DNACPR order was that the patient remained for ‘active treatment’. However, providing active treatment is not a reason not to consider and document what should happen in the event of a cardiac arrest.

Finally, there is growing legal and ethical concern [17] about the manner in which DNACPR decisions are approached, with decisions often not discussed or communicated effectively to patients or their relatives.

**Figure 1:**
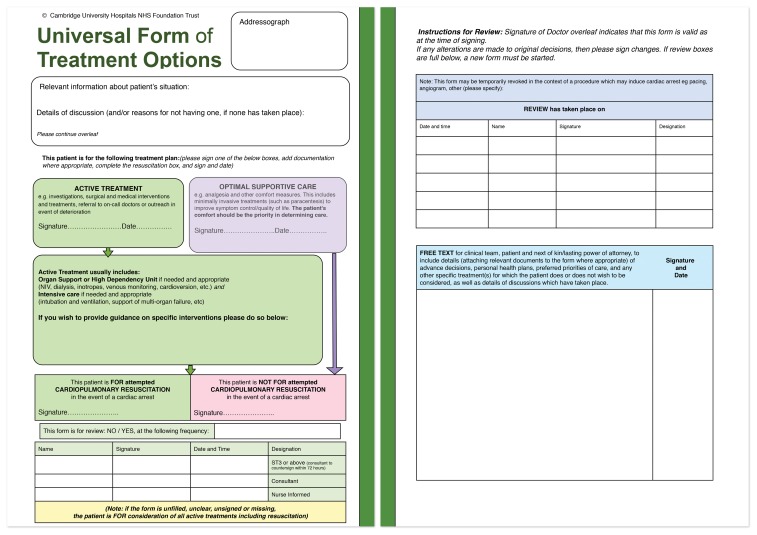
The Universal Form of Treatment Options (UFTO) version 21.

With the aim of improving communication about what care was desired and appropriate, we developed an alternative approach: The Universal Form of Treatment Options contextualizes the CPR decision within overall treatment plans, and is completed on every medical in-patient (UFTO - Figure 1). While alternative approaches have previously been developed, [18], [19] they have not been applied universally, nor their impact on patient care assessed.

Using a mixed-methods approach we sought to comprehensively evaluate the impact of the UFTO: in relation to clinical decision-making, patient safety and ward-based practice.

## Methods

### Summary

The protocol for this trial and supporting STROBE checklist are available as supporting information along with the appendix which contains details of the amendments; see Checklist S1, Protocol S1, and Appendix S1.

The trial was registered with ISRCTN- registration number 85474986 and the UK Comprehensive Research Network Portfolio- registration number 7932.

Ethics approval was obtained from the Norfolk Research Ethics Committee. The intervention was the introduction of a new approach to resuscitation decisions at ward level, and the ethics committee agreed that individual patient consent was not required for the introduction of the UFTO. Written patient consent was obtained for participating in interviews.

The UFTO was developed iteratively in collaboration with patients, doctors, nurses and resuscitation officers. The process included 20 semi-structured interviews, 6 focus groups with senior and junior nurses, senior and junior doctors from different clinical settings, and patients, and behavioral economist advice. Specific features of the form include completion of resuscitation status for all patients (in contrast with the often ad hoc DNACPR decision-making), and a focus on treatments to be given rather than withheld: in particular there was a distinction drawn between whether active treatment (with the emphasis on attempted cure) or supportive care (with the emphasis on symptom relief) was in the patient’s best interest. An accompanying patient information leaflet was also developed (Appendix S1).

A prospective mixed methods before-and-after study was carried out in a 480 bed acute hospital on two wards. Three months (May-July 2010) of qualitative and quantitative baseline data was collected on current (DNACPR) practice. The DNACPR form was completed whenever a physician thought it appropriate, or at a patient’s request. A month-long UFTO education period (further details of implementation policy and associated training materials can be found at ufto.org) was followed by two months of bedding-in, then three months data collection on UFTO practice (Nov 2010–Jan 2011).

Contemporaneously, a sample of patients with DNACPR orders from non-intervention wards was assessed.

#### Qualitative data collection

Face-to-face semi-structured interviews took place with all consultants and a purposive selection of nurses and junior doctors. Direct observation was undertaken on the participating wards both before and after use of the UFTO became routine practice to contextualize the interview data. Transcribed interviews together with field-notes were coded descriptively and thematically, providing the basis for a framework approach to analysis [20], [21].

#### Quantitative data collection

All patients in whom a decision not to attempt cardiopulmonary resuscitation was made during the study period (May-July 2010 and Nov 2010–Jan 2011) were eligible for inclusion. Those <18 years old or with an admission of <24 hours were excluded. Those who were determined to be for palliative care only within 72 hours of admission were initially excluded (Table 1), but were re-included in subsequent analysis to address possible confounding.

**Table 1:** Exclusions from dataset on study wards during DNACPR and UFTO periods.

	DNACPR period	UFTO period
Total included admissions	513	520
Missing notes	1	2
Excluded because length of stay <24 hrs	9	13
Excluded because age <18 yrs	2	3
Other Exclusions	1	3
Total non-palliative care exclusions	13	21
Palliative/Optimal Supportive Care initially excluded, reincluded in subsequent analysis	5	21

Abbreviations: DNACPR: Do Not Attempt Cardiopulmonary Resuscitation.

UFTO: Universal Form of Treatment Options.

Data were also collected contemporaneously on two case control groups: 1) patients remaining for resuscitation (every 7^th^ admission on the study wards); 2) patients from non-study wards, which had an electronically recorded DNACPR decision.

We collected baseline demographic and hospital data, along with a modified early warning score (MEWS) [22]–[24] on admission and Charlson co-morbidity scores [25].

**Figure 2:**
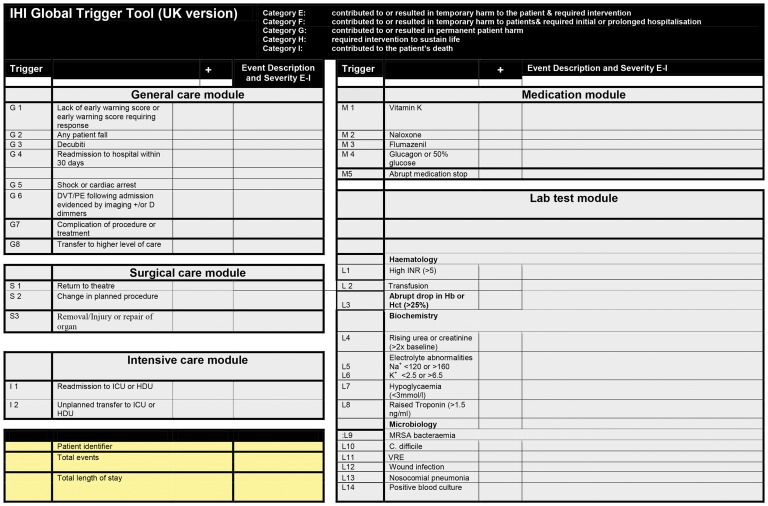
The United Kingdom Version of the Institute for Healthcare Improvement Global Trigger Tool (GTT).

The Institute for Healthcare Improvement Global Trigger Tool (GTT- Figure 2) [26], [27] was used to as a validated method of assessing rate, severity, and preventability of harms. Patient case notes were reviewed in a standardized way and in a random order, to identify predefined ‘triggers‘ such as a hospital acquired pneumonia or an early warning score requiring action, which may be an indication that harm has occurred.

Using preselected modules of GTT, the presence of any of the 29 triggers was recorded; no assessment was made at this stage of association with harm. A short paragraph describing each trigger event was inputted into a database. Multiple triggers could be recorded for an individual patient.

Blinded physician reviewers independently reviewed the information on the GTT triggers making a determination of presence, severity and preventability of harm. Severity was classified using the index of the National Coordinating Council for Medication Error Reporting and Prevention (NCC MERP): category E: temporary harms requiring intervention; category F: temporary harms requiring initial or prolonged hospitalization; category G: permanent harms; category H:life- threatening harms; and category I: harms causing or contributing to death. Preventability of harm was rated using a Likert scale with scores from 1 = definitely not preventable to 4 = definitely preventable.

Where reviewers did not agree about whether an event constituted a harm, the case was discussed and agreement reached. Although the GTT was initially intended as a quality improvement tool, the methodology for using it remains the same in the context of evaluating an intervention with contemporaneous case controls.

### Statistics

#### Sample size

The primary endpoint was timely (within 4 hours) referrals of patients with an Early Warning Score (EWS) of greater than 3. The power calculation was constructed assuming a two-sided Fisher’s exact test would be performed at the 5% significance level, on a EWS outcome representing the proportion of patients inappropriately managed. A sample size of 108 individuals with ‘Not for CPR’ orders per group was considered to provide 80% power to detect an absolute difference of 20% between the UFTO and DNACPR groups in the proportion of patients inappropriately managed (as defined by the EWS). It was anticipated, using preliminary data, that this number of patients would be admitted onto the study wards in a 3 month period.

The results we report here refer primarily to the secondary outcome measure of harms sustained by patients, as measured by the GTT. We have therefore applied a higher statistical stringency of 2% for significance.

### Analysis

#### Qualitative data

A preliminary set of codes from a sample was agreed upon to ensure that they were sufficiently reliable and unambiguous. During analysis further refinement allowed for the identification and inclusion of emergent themes as well as those drawn from relevant literature. These codes were subsequently used for mapping and interpretation of the key comparative themes between the DNACPR and UFTO phases including recognition of atypical cases. Discussion between clinical and anthropological authors ensured that clinical experience could inform the contextualization and interpretation of results.

#### Quantitative data

UFTO and DNACPR groups were compared by calculating the absolute rate difference of GTT harms between them. Patient characteristics were compared between groups using Fisher’s Exact test for all categorical variables and the Mann-Whitney test for all continuous variables except age, for which an independent samples t-test was used.

The frequency distribution of the type of harms, and the severity and preventability of harms was also tabulated for each group. The tables had low expected counts so the analysis focused on comparing proportions of serious harms and proportions of preventable harms using Fisher’s Exact test.

A Poisson regression model was fitted to the number of harms data to evaluate the effect of group (UFTO or DNACPR) on number of harms after adjusting for possible confounders.

As a sensitivity analysis, negative-binomial regression models were also fitted to account for any over-dispersion in the data. A log-transformed offset term was included for hospital length of stay, to adjust for differences in periods of observation across patients.

To address a known confounding factor, the effect of including palliative care patients in the analysis was tested using the same statistical methods by re-including them into the dataset.

Additional assessments, using the same analysis, were conducted on the two contemporaneous case control groups.

R software [28] and SPSS version 18 [29] were used for analyses. A 2% significance level was used for the GTT because of multiplicity of outcomes; 5% significance levels were used elsewhere.

## Results

There were 530 admissions (with 13 exclusions) during the DNACPR period and 560 (with 21 exclusions) in the UFTO period.

### Patient Data

Table 2 shows a comparison of patient characteristics for patients in whom a decision not to attempt CPR was made. There were no significant differences at the 5% level.

**Table 2:** Comparison of characteristics of patients in whom a decision not to resuscitate was made in both groups.

	Group		
	DNACPR (n = 103)	UFTO (n = 118)	p–value
Age	Mean 82.5 (SD 9.39)	Mean 82.1 (SD 9.11)	0.77
Female gender	47 (46%)	53 (45%)	1.00
Respiratory Ward	60 (58%)	73 (62%)	0.68
Length of hospital stay (days)	Median 12.0 (IQR 22.0)	Median 12.0 (IQR 16.25)	0.86
Charlson comorbidity score	Median 2.0 (IQR 3.0)	Median 2.5 (IQR 3.0)	0.61
MEWS score	Median 2.0 (IQR 3.0)	Median 2.0 (IQR 3.0)	0.97

### Completion of Form

**Figure 3:**
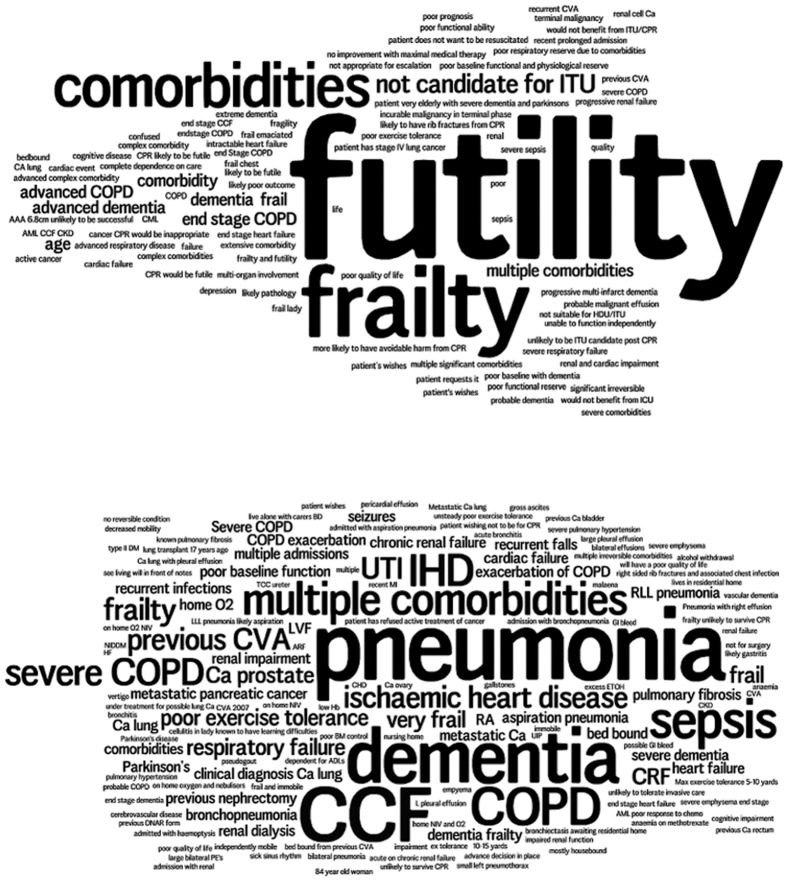
‘Word Clouds’ generated from summary text on forms of all patients not for cardiopulmonary resuscitation. 3a. Text taken from Do Not Attempt Cardiopulmonary Resuscitation orders. 3b. Text taken from Universal Form of Treatment Options.

The completion rate of the UFTO was 82%. The decision not to attempt CPR was documented in 108/517 patients (20.9%) in the DNACPR period and 139/539 (25.8%) in the UFTO period (Fisher’s Exact p-value = 0.07). ‘Word Clouds’ in which the size of the word represents the frequency of its use [30], [31] were generated from the summary texts written on both forms of patients in whom a decision not to resuscitate had been made (Figure 3). There was an increase in the number of patients who were recognized and documented as being for palliative or optimal supportive care within 72 hours of admission: 5/517 (1.0%) in the DNACPR group and 21/539 (3.9%) in the UFTO group (Fisher's Exact p-value = 0.002).

### Frequency of Referrals when Early Warning Scores >3

The frequency of EWS greater than 3 did not occur at the same rate as in the preliminary data, and there was no statistically significant difference in these results (Table 3).

**Table 3:** Non-GTT variables measured.

	DNACPR period A(May–July 2010)	UFTO period B(Nov 2010–Jan 2011)	Between group difference(95% CI)	P–value^§^
Discussion rate in those in whom adecision not to resuscitate was made(DNAR group n = 103; UFTO groupn = 118)	42/103 (41%)	41/118 (35%)	**6.0% (–6.7% to 18.6%)**	**0.40**
Early Warning Score (EWS) responsein those in whom a decision not toresuscitate was made (DNAR groupn = 103; UFTO group n = 118)	24/102 (24%)	19/117 (16%)	**7.3% (–3.3% to 18.0%)**	**0.23**
Length of hospital stay for thosenot for resuscitation (DNAR groupn = 103; UFTO group n = 118)	Median 12.0 (IQR 20.5)	Median 12.0 (IQR 15.75)	Median difference0.0 (–3.0 to 3.0)	0.86
Whole ward mortality	58/530 (11%)	71/560 (13%)	–1.7% (–5.6% to 2.1%)	0.40

### Rate of Harms - Global Trigger Tool (GTT)

Secondary reviewers had a concordance rate of 93.7% in establishing whether a documented GTT event constituted harm. There were 71 harms among 103 patients over 2048 patient-days in the DNACPR group, equating to 68.9 harms per 100 patient admissions (95% CI: 54.6 to 87.0), or 34.7 per 1000 patient days (Table 4). In comparison, there were 44 harms among 118 patients over 2021 patient-days for those not for attempted CPR (NFAR) in the UFTO group (hereafter referred to as UFTO/NFAR), equating to 37.3 harms per 100 patient admissions (95% CI: 27.7 to 50.1), or 21.8 per 1000 patient days. The rate difference per 100 patient admissions (DNACPR - UFTO/NFAR) was 31.6 harms (95% CI 12.2 to 51.1, p-value 0.001). The rate difference in harms per 1000 patient days (DNACPR - UFTO/NFAR) was 12.9 per 1000 patient-days (95% CI: 2.6 to 23.2, p-value 0.01).

**Table 4:** Global Trigger Tool Analysis on those patients in whom a decision not to attempt resuscitation was made (DNACPR group n = 103; UFTO group n  = 118).

	DNACPR period A (May–July 2010)	UFTO period B (Nov 2010–Jan 2011)	Between group difference(95% CI)	P–value^§^
Harm rate per 100 admissions	68.9	37.3	31.6 (12.2 to 51.1)	0.001
Harm rate per 1000 patient days	34.7	21.8	12.9 (2.6 to 23.2)	0.01
Harms contributing to patient death(categories H and I)	23/71 (32%)	4/44 (9.1%)	23.3% (7.8% to 36.1%)	0.006
Harms preventable on any level (categories 2–4)	66/71 (93%)	43/44 (98%)	–4.8% (–13.4% to 5.6%)	0.40

The Poisson regression and negative binomial models show a significant difference in rate of harm between the groups at the 5% level after adjusting for ward, age, gender, MEWS score and Charlson co-morbidity score.

### Severity, Type, and Preventability of Harms

There was a difference in the proportion of harms contributing to patient death in the two periods (p = 0.006) (Tables 4 and 5).

**Table 5:** Rating of Severity of Harms using the NCC MERP Index in DNACPR and UFTO groups.

	Group		Total
Severity	DNACPR	UFTO	
E	17	15	32
F	30	25	55
G	1	0	1
H	1	0	1
I	22	4	26
Total	71	44	115

Legend: NCC MERP Index.

Category E: Temporary harm to the patient and required interventionCategory F: Temporary harm to the patient and required initial or prolonged hospitalisation.

Category G: Permanent patient harm.

Category H: Intervention required to sustain life.

Category I: Patient death.

The frequency of each type of harm can be seen in Table 6. The categories which were most frequently associated with harms were ‘nosocomial pneumonia’ (determined by radiological changes) and ‘lack of Early Warning Score (EWS) or EWS requiring a response’. There was no significant difference in the preventability of harms (p = 0.40).

**Table 6:** The frequency of each type of harm for trigger categories within UFTO and DNACPR groups.

	Frequencies of harms per group	
Trigger	DNACPR	UFTO
L13 (Nosocomial pneumonia)	15 (21%)	10 (23%)
G1 (EWS requiring response)	10 (14%)	4 (9%)
G4 (Readmission within 30 days)	9 (13%)	6 (14%)
G3 (Decubiti)	6 (8%)	6 (14%)
M5 (Abrupt medication stop)	5 (7%)	1 (2%)
G7 (Complication of treatment)	4 (6%)	1 (2%)
G6 (DVT/PE)	4 (6%)	0
G2 (Fall)	3 (4%)	6 (14%)
M4 (Glucagon or 50% Dextrose)	3 (4%)	5 (11%)
L5 (Abnormal Na+)	3 (4%)	0
L3 (>25% drop in Hb)	2 (3%)	1 (2%)
L4 (Rising Urea or creatinine)	2 (3%)	1 (2%)
L6 (Abnormal K+)	2 (3%)	0
M2 (Naloxone administered)	1 (1%)	0
L1 (High INR)	1 (1%)	0
L8 (Raised Troponin)	1 (1%)	0
L7 (Hypoglycaemia)	0	2 (5%)
L2 (Transfusion)	0	1 (2%)
Total harms	71	44

### Inclusion of Palliative Care Patients

Because our intervention changed the number of patients excluded, we collected data on those patients identified as being for palliative care. When these patients were analyzed, the rate difference per 100 patient admissions (DNACPR - UFTO/NFR) was calculated to be 32.6 harms (95% CI: 14.4 to 50.8; p-value <0.001), or per 1000 patient-days the rate difference was 14.7 harms (95% CI: 5.0 to 24.4; p-value 0.003).

### Contemporaneous case Control Studies

In patients with DNACPR orders, taken from wards where the UFTO was not introduced, there was no significant change in rate of harms: 52 harms/100 admissions or 18 harms/1000 patient days in May–July 2010 versus 68 harms/100 admissions or 32 harms/1000 patient days in Nov 2010–Jan 2011 (95% CI: −26.9 to 58.9; p-value 0.47 and 95% CI: −4.1 to 32.4; p-value 0.13 respectively). Multivariate regression on these groups showed no significant difference in rate of harm between the UFTO and DNACPR periods at the 5% level, even after adjustment for ward, age, gender, MEWS score, and Charlson co-morbidity score.

There was no significant change in harms observed in a sample of patients remaining for resuscitation from the study wards in the same period (Table 7).

**Table 7:** Balancing measures of GTT in those patients for resuscitation (n = 60 in period A, n = 58 in period B) and on patients in whom a decision not to resuscitate was made on non–study wards in the same periods (n = 25 in period A, n = 25 in period B).

	DNACPR period A(May–July 2010)	UFTO period B(Nov 2010–Jan 2011)	Between group difference(95% CI)	P–value
Harms rate per 1000 patient daysin those for resuscitation	7.1	7.3	–0.2 (–9.6 to 9.3)	0.97
DNAR harms rate per 1000patient days in non–studywards	18	32	–14.2 (–32.4 to 4.1)	0.13

### Secondary End Points

There were no significant differences seen with discussion rates, mortality, or length of stay (Table 3).

### Interviews and Observation

Forty-seven interviews were conducted with nurses and physicians, the results of which were integrated with field-notes from prolonged periods of situated observation. The key themes derived from our adapted framework analysis comparing DNACPR and UFTO are summarized in Table 8 with illustrative quotations. We identified three main domains of care in which it was possible to compare the use and understanding of the original DNACPR with those of the UFTO. These were: Interdisciplinary communication; clarity and consistency; patient dignity and respect. In each one, interviewees contrasted use of the original DNACPR form with the UFTO, highlighting a range of advantages they associated with the new form.

**Table 8:** Key comparative themes emerging from interview accounts.

Domains of Care	DNACPR	UFTO (with illustrative quotation)	
Interdisciplinary communication,clarity andconsistency	Unequivocal,‘STOP’ sign	Sense of direction/forward planning	“basically made us question where we were going with the patient from the beginning.” (SPR) “It gives a plan; it makes the doctors do a plan for the patients so that you’re completely in the picture… as to who’s for resus, how far we’re going to go for active treatment, for escalation to ITU, that type of thing. And who isn’t for resus but they’re still for active treatment and are going to escalate, how far are we going to escalate” (Nurse)
Interdisciplinary communication,clarity andconsistency	Arbitrary, ad hoc,only at crisis point	Systematic	“everyone has to have one, so it is thought about at the time of admission… before it was if someone suddenly becomes poorly and then you think ‘Oh, were they for resus?’ and then you realise they are and then there’s all a bit of a hoo–ha about trying to change that quite quickly” (Nurse)
Interdisciplinary communication,clarity andconsistency	Marking out,‘special case’	Habitual, universal,routine	“with the UFTO because everybody gets one you kind of get into the habit of constantly thinking about it for everyone” (Junior Doctor)
Interdisciplinarycommunication,clarity andconsistency	Unofficial triage	General clinicalsummary	“If you’ve got all the information in one place rather than flicking through four weeks of admission… you know, that can only be a good thing for a patient.” (SPR)
Interdisciplinarycommunication,clarity andconsistency	Insidious	Open	“it has been a long time now since somebody has asked me about somebody who wasn’t for resuscitation whether we should be actively treating them. Because it quite clearly says” (Consultant)
Patient dignityand respect	Potentially negativeassociations forpatients/relatives	Normalising forpatients/relatives	“If you say everyone gets one it makes them feel better that it’s sort of part and parcel of coming in, and it’s not that we think they’re going to die” (Junior Doctor)
Patient dignityand respect	Negativeassociationsfor clinicians	Normalising forclinicians	“now I think because everyone has the UFTO it’s more like they’re for treatment whether or not for resus” (Junior Doctor)
Patient dignityand respect	Precipitatesevaluations offutility	Encouragesevaluations ofappropriate actions	“you know that there’s been a thought process, it’s not just some sort of arbitrary decision based upon the initial assessment of the patients’ chances” (Nurse)
Patient dignityand respect	Clinical discomfortwith decision	Clinical comfortwith decision	“I do find it more comfortable that I can say for ward level of care, antibiotics and things, but not for CPR…” (Consultant)
Patient dignityand respect	Stigma of formdiscouragesconversations withpatients andrelatives	Makes clinicians morecomfortable in theirdiscussions withpatients and relatives	“once you’ve explained it and you’ve shown them the form, they [a patient’s relatives] do feel happier.” (Junior Doctor)
Pragmatic details	Recognisable inan emergency	Recognisable inan emergency	“it’s something that, the same as DNACPRs, it’s somewhere that’s easily accessible, you can find it… you can see things quite easily and quickly” (Registrar)
Pragmatic details	Straightforward tocomplete – notdemanding on time	Straightforward tocomplete – takes alittle time but savesmore time later on	“you’re putting the effort in filling them in; so’s everybody else which makes your on–calls easier. Then, you know, that’s the kind of culture that perpetuates itself… it is more hard work filling in the forms, but it’s appropriate hard work. It’s not like it’s creating work, we should be considering DNACPR on all patients but it’s just not done.” (Registrar)
Pragmatic details	Permanent recordof a single clinicaldecision	Permanent recordof a range ofclinical decisions	“it’s also good because DNARs, yeh that’s fine it kind of says ‘if this person’s heart stops beating we’re not, you know, going to resuscitate them’ but it doesn’t give any other sort of advice about ‘if this patient deteriorates massively what’s our ceiling of care?’ … Especially when you’re on call and you don’t necessarily know what has been happening with the patient and the limits of treatment are. So if you’ve got something like that to be able to say “right, ok, they wouldn’t go to ITU”, that’s helpful. ” (Junior Doctor)

Prior to the introduction of the UFTO, completing DNACPR forms was not routine; they were initiated at *ad hoc* times and sometimes based on unsystematic criteria. One major concern raised about the introduction of the new form was the likely increase in workload. This referred not only to UFTO completion, but also that there might be an exponential rise in the number of discussions with patients and their relatives. Once the UFTO was embedded, however, clinicians reported there was a reduction in negative associations for patients who were not for CPR because of the routine and universal application of the UFTO. Staff commented that use of the UFTO both initiated and recorded forward planning, giving them a much better “sense of direction” about the care of the patient. Increased clarity of goals resulted in better communication between clinicians, particularly out of hours.

These qualitative insights corroborate the quantitative findings; the introduction of the UFTO was perceived to make a positive global difference to how staff delivered care to many patients. Frequently this was described in general terms instead of ways in which it influenced specific treatment decisions or interventions. Rather than being able to link observations and interview data to particular instances or types of harm reduction, the qualitative findings suggest that the UFTO shifted ward practices in range of inter-related ways.

## Discussion

### Clinical Impact

#### Reduction in harm

Use of the UFTO was associated with an appreciable decrease in both frequency and severity of harms in patients for whom a decision not to attempt CPR was made. The characteristics of the patients studied, in terms of age, co-morbidities, and sickness at admission were similar, and the reduction in rates of harm was maintained after palliative care patients were re-included in the analysis. There was no such reduction in harms in patients with DNACPR orders on other wards during the same time-period, or for patients remaining for CPR, suggesting that the change we observed was due to the use of the UFTO, and not seasonal variation or hospital-wide safety improvements. Accepting that we were looking at a group of patients who have worse outcomes than the standard hospital population, it is worth noting that no previous study of any initiative aimed at improving patient safety (as measured with the GTT) has shown such a profound effect, and that while alternative approaches to recording DNACPR decisions have previously been developed they have not been rigorously assessed for impact upon patient care [18], [19].

The mechanism for this quantifiable reduction in harm can be partly understood from existing literature which demonstrates that standard DNACPR orders are often misinterpreted, leading to treatments being withheld [5]–[11], and by drawing on the qualitative findings. Nurses and doctors explicitly reported that they felt they were able to provide better care with the UFTO: clinicians’ attitudes towards patients were re-orientated by focusing on the primary decision between active or supportive treatment, and treatments to be given, rather than a treatment to be withheld; the removal of the stigmatizing, negative effect of the red DNACPR order [32] led to less distinction between patients who were for and not for CPR. Nurses and doctors unanimously requested to continue using the form despite their recognition that it added to the workload on admission.

#### Use of the UFTO

Use of the UFTO rapidly became habitual, with over 80% completion. Doctors and nurses incorporated the UFTO into their handovers, and found it useful when reviewing a patient out-of-hours. Word clouds (Figure 3) demonstrate the changed nature of what was written about patients. From a predominant use of the word “futility” on the DNACPR forms, there was a shift to document diagnoses on the UFTO. Interviews and observations suggested that the new form at the front of the notes acted as a summary of the patient’s condition and which treatments would be appropriate, while continuing to document CPR status. Use of the UFTO led to a significant increase in the number of patients identified early as requiring palliative or optimal supportive care, with no change in mortality. It is possible that this early recognition allowed better palliative care to be delivered. Several tools exist [33] to alert doctors to when a shift in goals of care might be appropriate, but the benefit of the UFTO is that it will do so universally.

Perhaps surprisingly, given the universal nature of the UFTO, there was no significant difference in the proportion of patients in whom a decision was made not to attempt CPR. There was also no significant change in other patient characteristics (age, co-morbidity, sickness on admission), suggesting that the UFTO did not affect the threshold of the CPR decision. Physicians reported that they felt more comfortable ‘making’ a patient ‘not for resuscitation’, while simultaneously documenting that a patient was for ‘active treatment’. The benefit of mandating decision-making about CPR is that there are likely to be fewer “inappropriate” resuscitation attempts, in particular when patients might not want such an attempt.

#### Discussions

The number of documented discussions with patients did not increase, despite all patients being given a leaflet encouraging them to discuss treatment options with their physicians. This finding is consistent with a previous study [34]. Although several studies have suggested that patients want to have discussions about CPR, they have significant selection bias [35], [36]. Doctors using the UFTO reported that conversations with patients were ‘easier’ but it is hard to quantify this, or know whether this benefits patients. Further work is needed to investigate whether patients would like to be actively involved in UFTO completion, and if patient capacity should be included in the UFTO.

#### Limitations

Although we have addressed several limitations, this remains a ‘before and after’ study with contemporaneous case controls. We tried to minimize the Hawthorne effect by interviewing clinicians in both arms of the study, and having a two-month ‘bedding in’ period before assessing the UFTO. We did not conduct a DNACPR education package before the DNACPR period, because we wanted to compare the UFTO with standard practice, but recognize that the effect we saw might in part relate to education provided with the introduction of the UFTO. The intervention may have changed the population we were studying, by increasing the proportion of patients who were identified as being for optimal supportive care. However, we have re-analyzed our data with the palliative patients included, with consistent results. We were not powered to determine whether the intervention affected mortality or length of stay, but these are two outcomes which would be interesting to assess in a larger study. Finally, we were unable to interview patients in this study. Our ethics approval was to interview patients with whom resuscitation decisions had been discussed; the frequency of these discussions was low, and even when they had been documented, patients often did not remember having such discussions. Further research is therefore needed to understand the patient and family perspective.

## Conclusion

The decision not to attempt cardiopulmonary resuscitation is often a reasonable and ethically sound one, either because of patient choice, or because attempting resuscitation would deprive a patient of dignity in their death or risk causing more harm than benefit. Unfortunately there is mounting evidence that those with DNACPR orders also receive inadequate treatment.

By changing the approach to resuscitation decisions – contextualizing resuscitation amongst other treatments and ensuring that documentation is universal - a major shift was seen in the behavior of nursing and medical staff. This work indicates that an alternative approach, delivered by a simple form, has the ability to improve care for this group of vulnerable patients.

## Supporting Information

Protocol S1 **Trial Protocol.**
(DOCX)

Checklist S1 **STROBE Checklist.**
(DOC)

Appendix S1 **Online appendix including details of Standard Operating Procedures, protocol amendments, Patient Information leaflet “Talking with your doctor” and detailed breakdown of quantitative and qualitative data.**
(DOCX)
