# Supplementary material for: The Universal Form of Treatment Options (UFTO) as an Alternative to Do Not Attempt Cardiopulmonary Resuscitation (DNACPR) Orders: A Mixed Methods Evaluation of the Effects on Clinical Practice and Patient Care
Source: PLoS One. 2013 Sep 4;8(9):e70977. doi: 10.1371/journal.pone.0070977 (PMC3762818; doi:10.1371/journal.pone.0070977)
Supplement: Protocol S1 — Trial Protocol. (DOCX) [file pone.0070977.s001.docx]

**Protocol S1. Trial Protocol**

Cambridge UFTO Research Group.

Clinical Study Protocol

Protocol Number PB-PG-0808-17218

**Study Title**: A Universal Form of Treatment Options (UFTO): A study to evaluate the UFTO as an alternative to the DNACPR

Protocol Version: 2.3

**Chief investigator**: Dr. Zoe Fritz

**Co-investigators:** Dr. Jonathan Fuld, Dr. Simon Cohn, Dr. Chris Palmer, Dr. Gary Barton, Dr Clare Laroche, Professor Tony Hope, Mrs. Rebekah Lay

Research Assistants: Jude Frankau and Alexandra Malyon

**Investigator’s address:**

UFTO Project Box 148, Addenbrookes Hospital, Cambridge CB2 0QQ

**Study Location:**

Addenbrooke’s Hospital, Cambridge and the West Suffolk Hospital, Bury St Edmunds.

Telephone: 01223 274597

**Study Sponsor:**

Mr Stephen Kelleher, Research and Development Manager, Box 277, Addenbrooke’s Hospital, Cambridge CB 2 0QQ

Sponsor Medical Expert: not applicable

**Medical Contact on site:**

The medical site manager, tel. 01223 245151 bleep 1560 707

Study Synopsis

| Title of clinical trial | A Universal Form of Treatment Options (UFTO): A study to evaluate the UFTO as an alternative to the DNACPR |
| --- | --- |
| Sponsor name | Addenbrooke’s Hospital |
| Medical condition or disease under investigation | All medical conditions |
| Purpose of clinical trial | To assess the UFTO as an alternative to the DNACPR. The UFTO will be filled in on all patients, and encourages the documentation of treatment decisions including resuscitation. We hypothesise that it will standardise physicians approach to resuscitation while respecting clinical judgement and patient choice. |
| Primary objective | To assess whether the UFTO improves the number of referrals in response to MEWS (modified early warning system) ≥ 3 in those patients who are not for resuscitation. |
| Secondary objective (s) | We will assess, along with legal, health economic and ethical implications, whether the form:   - Improves care for those not for resuscitation as measured by referral to specialities and other escalating care (e.g. HDU / ICU). - Reduces the number of patients who remain inappropriately for resuscitation. - Improves understanding in all groups about the appropriate implications of the decision not to resuscitate. - Creates a positive framework for discussions of treatment options with patients and their families   . |
| Study Design | Before and after study |
| Study Endpoints | Discharge from hospital, or transfer to another ward |
| Sample Size | 108 patients who are not for resuscitation in each group |
| Summary of eligibility criteria | All patients over 18 years of age, excluding those for palliative care on admission or with an admission of less then 24 hrs |
| Investigational medicinal product and dosage | No medicinal product included |
| Active comparator product(s) | DNACPR versus UFTO |
| Procedures: Screening & enrolment | All patients admitted to selected clinical areas are eligible |
| End of Study | Patient discharge or transfer to another ward. 30 day mortality will be assessed. |

**AMENDMENT TO PROTOCOL- NEW DATASET**

Following initial analysis of data from this study it was found that EWS scores greater than 3 did not occur as frequently as had been found in the preliminary data, therefore there was no statistical significance in these results. The collection of an additional dataset was then proposed and ethics approval obtained for an amendment to allow new data to be obtained using the IHI Global Trigger Tool. This tool was not in widespread use in the UK when the initial study was planned.

**Aim**

To assess rates of harm, as measured by the IHI Global Trigger Tool, in all patients ‘not for resuscitation’ during both the DNAR periods and following the introduction of the UFTO and to assess whether the UFTO was associated with reduced rates of harm in those ‘not for resuscitation’.

**Endpoint**

The primary endpoint was rate of harms per 1000 patient bed days as determined by the IHI Global Trigger Tool methodology. This was assessed in all patients ‘not for resuscitation’ in both the DNAR and UFTO period. The rate of harms per 100 patient admissions was also calculated within each patient group.

Since this was not our initial end point, a more stringent criteria of 0.02 will be considered statistically significant.

**Contemporaneous case controls**

Additional analysis were carried out on two contemporaneous case control groups.

1. A selection of patients on the studied wards who remained for resuscitation during the same period.
2. Those ‘not for resuscitation’ on wards where the intervention, the UFTO was not introduced during the same period.

**Statistical Analysis**

The primary endpoint was the rate of harms per 1000 patient bed days in those patients ‘not for resuscitation’, calculated separately for the group of patients with a DNACPR order and those receiving UFTO. The rate of harms per 100 patient admissions was also calculated within each patient group. The UFTO and DNACPR groups were compared by calculating the absolute rate difference between the groups with a corresponding 95% confidence interval. A stratified analysis was then performed by calculating the rate differences within each ward group. Patient characteristics were compared between groups using the Fisher’s Exact test for all categorical variables and the Mann-Whitney test for all continuous variables except age, for which an independent samples t-test was used. This was because all continuous variables except age had non-normal distributions.

The frequency distribution of the type of harms, and the severity and preventability of harms was tabulated for each group. The chi-squared test for trend was utilized where possible to statistically compare between the groups. However, in the case of tables with low expected counts, Fisher’s Exact tests were used.

Difference in proportions were calculated to compare the number of harms contributing to patient death and the number of harms preventable at any level between groups, with corresponding 95% confidence intervals calculated using a recommended method (Newcombe’s method) [1]. The same method was used to compare discussion rates, EWS response and mortality between groups.

A Poisson regression model was fitted to the number of harms data to evaluate the effect of group (UFTO/NFR or DNACPR) on number of harms after adjusting for possible confounders. For the first model, only the group variable was included as an explanatory variable in the model; and then ward was also added; and finally, a model was fitted including all covariates of interest: ward, gender, Charlson co-morbidity score, MEWS score and age at admission. For all models, a log-transformed offset term was included for hospital length of stay, to adjust for differences in periods of observation across patients.

As a sensitivity analysis, negative-binomial regression models were also fitted to account for any over-dispersion in the data. As before, a log-transformed offset term was included for hospital length of stay, to adjust for differences in periods of observation across patients.

To determine the effect of including palliative care patients in the analysis, exactly the same statistical methods were employed as above but with the addition of the palliative care patients.

R software version 2.15.0 [2] was used for most analyses, except SPSS software version 18 [3] was used for data manipulation and to calculate summary statistics and p-values when comparing patient characteristics between groups.

**The IHI Global Trigger Tool Methodology**

The IHI Global Trigger Tool (IHI GTT) was developed by the Institute for Healthcare Improvement (IHI) to identify “harm” which has occurred to patients in hospital. A UK version has become widely used since 2009. The GTT has a series of objective “triggers”  - for example a fall, or a lack of early or EWS requiring response.

Primary review will be conducted in accordance with the IHI guidelines in a standardised fashion (outlined in a Standard Operating Procedure in an appendix to this protocol) and in 20 minutes or less, by one of two reviewers. Primary reviewers, one research nurse and one junior physician, will review case notes in a random order. Using predetermined selected modules of the UK Global Trigger Tool, any of the 29 triggers met (not just those which they think might be associated with a harm) will be recorded into a filemaker database. Primary reviewers will write a paragraph or two describing each trigger event, no determination of the presence of harm will be made at this stage. Multiple triggers can be recorded for an individual patient. This first 20 sets of notes will be reviewed by both primary reviewers to establish inter-rater reliability and standardization of the review process.

Secondary Consultant physician reviewers will then review the information prepared by primary reviewers. The secondary reviewers will be blinded to demographics, dates of admission and discharge, ward, and whether the patient was in the DNAR or UFTO group.

To maximise concordance, focus specifically on what occurred during the admission, and to minimise confounding, some additional criteria not included in the standardised GTT were predetermined before rating of harm, again outlined in more detail in the SOP for GTT review in an appendix to this protocol.

Secondary reviewers will independently assess each blinded record, determining the presence of a harm associated with a trigger. Where a harm is present, the severity and preventibility will also be recorded. Severity will be classified using the index of the National Coordinating Council for Medication Error Reporting and Prevention (NCC MERP) This gives five categories of harm- category E: temporary harms requiring intervention; category F: temporary harms

requiring initial or prolonged hospitalization; category G: permanent harms; category H:life- threatening harms; and category I :harms causing or contributing to death.

A Likert scale with scores ranging from 1 for “definitely not preventable” to 4 for “definitely preventable” will be used to evaluate preventability

Cases in which physician reviewers disagree will be discussed, and consensus reached; where this is not possible, a third opinion will be obtained. Inter-rater reliability on the presence of aharm will be calculated from pre-discussion ratings.

# References

# [1] Altman, D.G., Machin, D., Bryant, T.N., and Gardner, M.J. (2000). *Statistics with Confidence: Second Edition*, pages 45-55 (Chapter 6), BMJ books.

# [2] R Development Core Team (2012). R: A language and environment for statistical computing. R Foundation for Statistical Computing, Vienna, Austria. ISBN 3-900051-07-0, URL <http://www.R-project.org/>.

[3] **SPSS/PASW for Windows, Rel. 18.0.3. 2010. Chicago: SPSS Inc.**

Introduction

Background

Do Not Attempt Resuscitation (DNACPR) orders are likely to affect all people directly or indirectly: 68% of the population dies in hospital(1) and 80% of those die with DNACPR forms in place(2). DNACPR forms do not only affect those who are dying: many patients with DNACPR forms are discharged home(3).

DNACPR forms instruct the clinical team what to do in the event of a cardiac or respiratory arrest, and are not meant to affect other aspects of care(4).

They are filled in when a physician believes that a patient would not benefit from attempted resuscitation, or at a patient’s request. There is no legal obligation to tell a patient that such a form has been completed for them and a patient has no legal right to demand its removal.

In clinical practice we observed multiple problems with the current use of DNACPR forms.

• A reluctance amongst colleagues to refer patients with DNACPR orders for more intensive treatments;

• Hesitation before filling out a DNACPR form on patients, for fear it might have farther reaching impacts;

• Lack of communication between teams clarifying extent of treatment that was appropriate

• Variability in consultant practice for filling out DNACPR forms, resulting in the unethical situation where the decision concerning resuscitation for the individual patient was often made on an ad hoc basis. This can result in patients having resuscitation inappropriately attempted, with resulting discomfort, lack of dignity, and associated health costs

We investigated these further with preliminary studies and literature reviews as detailed below, and have summarised the ethical challenges that these problems pose in a paper in the Journal of Medical Ethics (5).

Patients, carers and health care professionals are uncomfortable discussing DNACPRs(6-8), although studies have also shown that, when asked, patients would like to have these conversations, despite the associated distress(9, 10).

Significant variability exists in the manner that DNACPR is approached and in the frequency of DNACPRs being implemented(11). Local data supports this (annex 4). This variability increases the difficulty of discussing DNACPR.

There is evidence that DNACPRs are misinterpreted to mean that treatments other than resuscitation should be withheld or diminished (12-14). A recent questionnaire carried out in our hospital amongst 50 doctors and 35 nurses confirmed this (15).

Consistent with this studies have shown that DNACPRs alter patient care; Beach et al (16) carried out a survey of doctors on hypothetical patients, and demonstrated that the presence of a DNACPR had an effect on decision making.

Those with DNACPRs have less intensive care (17, 18) and increased mortality (19, 20)

Alternatives to a DNACPR have been created, and their impact on documentation (21, 22), rate of CPR attempts and ventilation (23) assessed.

Advanced care plans have been developed to improve end-of-life care for palliative patients (24), but to our knowledge, a Universal Form for Treatment Options (which would be filled out on every patient, and include multiple treatment options) has never been developed, nor has the impact of any alternative to the DNACPR been assessed for changes in patient care and survival outcomes.

We interviewed 19 senior medical and nursing members of the hospital from all adult specialities (summary of interviews: annex 4) and had a consultative meeting with 2 lay panels (minutes: annex 4) to reach consensus on an alternative to the DNACPR form. We then had meetings with a physician panel, two nurse panels, and a further patients panel. The form was assessed with volunteer doctors and an actress to test out use. The result of this consultative process is the Universal Form for Treatment Options (UFTO) It was then piloted for two weeks, and further assessed with a cohort study at the West Suffolk.

The Investigators

Zoë Fritz is a Specialist Registrar in Acute Medicine and Intensive Care, and a board member of the Institute for Medical Ethics. Her initial observations, literature search and preliminary studies led to the formulation of the research question. Jonathan Fuld is a consultant in Acute and Respiratory Medicine. He is the local clinical lead for a large quality improvement initiative and has experience managing randomised controlled trials. Simon Cohn is a Medical Anthropologist with extensive research experience of patient and professional perspectives. He will manage the qualitative components of the project. Chris Palmer is Director of the Centre for Applied Medical Statistics, Cambridge and provides expertise in research design and data analysis. Clare Laroche is a Consultant in Respiratory Medicine and Clinical Director for Medicine at West Suffolk Hospital. She has an MD and has previously held two large NHS Executive research grants. Tony Hope holds the chair of Medical Ethics at Oxford; he will lead on the ethical implications of the project. Garry Barton is a Lecturer in Health Economics, and has conducted economic evaluations within a number of pragmatic RCTs. Rebekah Ley is Assistant Director Medico-Legal and Patient Experience at Cambridge University Hospital Hospitals and brings legal and ethical expertise. Jude Frankau is an early career social anthropologist, with research interests in shared decision-making in medicine. Alex Malyon is a research nurse with specific expertise caring for the critically ill.

12 lay individuals comprise an advisory group. They come from varied backgrounds; all have had experiences with resuscitation decisions either regarding themselves or a loved one.

Trial Aims and Objectives

Trial Aims:

The aim of this trial is to evaluate the UFTO, as an alternative to DNACPR, in reducing inequities of care resulting from current approaches to resuscitation decisions and documentation.

We hypothesise that use of the UFTO will

- contribute to a standardisation in doctors’ approaches to DNACPR decisions, whilst allowing patients’ individual choices to be respected;
- increase the number of discussions (both patient-initiated and doctor-initiated) which take place between doctors and patients about treatment decisions;
- encourage the documentation of clear treatment plans on what to do in the event of a patient’s sudden deterioration;
- reduce the number of patients who die in hospital without clear guidance as to appropriateness of resuscitation attempts from currently close to 20% to close to 0%. We hypothesise that this will result in fewer patients inappropriately remaining for attempted resuscitation;
- remove inequalities of treatment currently associated with a DNACPR form;
- increase satisfaction with and frequency of discussions pertaining to resuscitation decisions. Such benefits should be demonstrable in all groups;
- serve as the basis for general guidelines around the resuscitation discussion, so that doctors feel more supported when raising the topic with patients and relatives, who in turn experience it as an appropriate and necessary part of their overall care.

We aim to evaluate this using both quantitative and qualitative research methods.

*Primary objective*

To assess whether the UFTO improves the number of timely referrals in response to MEWS (modified early warning system)≥ 3 in those patients who are documented as not for resuscitation.

[*MEWS* is an objective physiological scoring system, based on pulse, blood pressure, etc. (detailed below, under section ‘Quantitative Analysis’)

*Timely* is defined as within 4 hours of the MEWS score being recorded

*Referrals* include referrals to the outreach team and the doctors.]

***Secondary objectives***

We will assess, along with legal, health economic and ethical implications, whether the form:

Increases care for those documented as not for resuscitation, but for active treatment (defined as active treatment in the UFTO group, and no statement of ceiling of care in the DNACPR group). This will be measured by:

- 1. review by other specialities
  2. referral and acceptance to escalating care (e.g. HDU / ICU)
  3. radiological investigation
  4. prescription of “expensive” drugs (where “expensive” is from a predefined list supplied by pharmacy);

Specifically, we will assess whether introduction of the UFTO alters *in isolation and as a composite endpoint* the above mentioned factors.

- Alters length of stay or in-hospital mortality;
- Improves care towards the end of life by encouraging advance care planning;
- Reduces the number of patients who remain inappropriately for resuscitation as judged by total numbers;
- Improves understanding in all groups about the appropriate implications of the decision not to resuscitate, as assessed by qualitative interviews and questionnaires and ethnographic research;
- Creates a positive framework for discussions of treatment options with patients and their families, as assessed by qualitative interviews and questionnaires and ethnographic research;
- To map the current decision-making processes around resuscitation: both explicit and implicit factors, across all groups
- To assess all groups’ attitudes and feelings about the old DNACPR and new UFTO, and compare their differing perspectives
- To assess the ethical problems faced by professional staff in using the conventional DNACPR form and the new UFTO
- To establish what elements of experience from each differing perspective are felt to be associated with the question of resuscitation in order to recognise the ways it has meaning in terms of the overall expectations of care
- To learn from outcome data of the above to inform an optimised approach to resuscitation decisions framed by the UFTO.

Trial Design

EVALUATION OF THE UFTO

The form to be trialled has been developed as described in the introduction

Study Summary

A before and after study will be carried out on the respiratory and general medicine ward, and General Medical/endocrinology and elderly care ward (G4) at the West Suffolk Hospital. Patients under 18 or for palliative care only on admission or who have an admission of less the 24 hours will be excluded.

We will prospectively collect the previously described data for a 3 month period prior to introduction of the UFTO form. Education of clinical teams will then be carried out by members of the research team.

Following a “bedding in” period of a month the UFTO form will be introduced throughout the clinical area. During this period, we will administer questionnaires to consenting nurses, doctors, patients and relatives who have had a discussion about resuscitation. We will interview a representative sample of nurses, doctors and patients about their understanding of the UFTO, and their views on the education we provided.

Following introduction of the UFTO the quantitative and qualitative data set will be collected for up to a further 3 months, or as long as it takes to collect a sufficient sample size for statistical significance ( at least 108 patients not for resuscitation).

**Education Package**

“Education of teams” will consist of a standardised presentation about using the UFTO, posters for clinical areas and information leaflets for staff (tailored for nurses and doctors to read after the education session. Members of the study team will answer any further questions in person or by email after the education period. A mini site within the hospital intranet will be created to support individuals using the new form.

**Sample size determination**

The primary endpoint for this study was the number of timely referrals of patients with an Early Warning Score (EWS) of greater than 3. The power calculation was constructed assuming a two-sided Fisher's exact test will be performed at the 5% significance level, on a EWS outcome representing the proportion of patients inappropriately managed. Those patients who had a high MEWS score above 3 and who were not referred on at least one out of four measurement occasions were considered to have been inappropriately managed. A sample size of 108 individuals with 'Not for Resuscitation' orders per group provides 80% power to detect an absolute difference of 20% between the UFTO and DNAR groups in the proportion of patients inappropriately managed (as defined by the EWS). It was anticipated, using preliminary admission data, that this number of patients would be admitted onto the study wards in a 3 month period.

**Number of Subjects**

Two clinical areas will be trialed: A respiratory and general medical ward and a care of the elderly/diabetes and endocrinology ward. The two wards combined have an average of 8 admissions a day, which should allow us to obtain the 108 patients not for resuscitation admissions necessary, estimating that one fifth of patients have a decision not to attempt resuscitation. We are powered to 108 patients in the subgroup of interest; it is possible that the overall sample sizes will be larger or smaller depending on rate of decisions about resuscitation. Staff and relatives will also be involved in the research, taking part in interviews, questionnaires, and ethnographic research.

**Eligibility criteria and screening evaluation**

All patients admitted to the two wards will be eligible for the study.

Those under 18, with an admission of less than 24 hours or for palliative care only on admission will be excluded.

**Study duration**

Patients will be involved in the study until their discharge or transfer to another ward. The end of the trial will occur when we have 108 patients who are not for resuscitation in each group (estimated at 720 patients total in each group, total sample size 1440).

**Data Collection**

Data will be collected from the medical and nursing notes by ZF, JMF and AM on

- Patient demographics (baseline observations and MEWS, age, gender, diagnosis, comorbidities, number of medications on admission),
- Dates of admission, and discharge/death to determine length and outcome of stay,
- Dates of initiation of palliative care, when applicable
- Ward
- DNACPR/UFTO completed (date, discussion documented, details on form)
- MEWS scores over 3 documented, with date and score.
- Appropriate (as defined by MEWS Score) and timely (within 4 hours) referral to Outreach services
- Referral to ICU/HDU/Out-of-hours medical escalation / medical speciality referral
- Number and type of radiological investigations
- Expensive drugs used
- Date and place of discharge
- Details of discussions / decisions in notes regarding end of life planning, resuscitation decisions, etc. (staff details will also be anonymised).

Data collected from the notes about number of resuscitation calls, and outcomes from these calls will be correlated with the information collected routinely by the resuscitation officers, to ensure accuracy of data.

The hospital Information team will provide data on patient mortality; we will calculate rate of mortality at 30 days post admission for all patients admitted during the study period.

**Logistics of Data collection**

**Full Data set**

Secretaries on the relevant wards will be asked to put notes of all patients discharged in a box for us to look at before the notes are sent back to medical records. Patient identification details will be put into a password protected spreadsheet, along with their study number. The patient details and study number will be inputted into an encrypted database. Anonymised data as listed above will be inputted into Filemaker (database software), on a shared, password protected, hospital drive. A record of all patients admitted to the wards during our study period will be accessed via the electronic medical records system at the hospital. This list will be cross checked with the one we have generated, and any missing patients will have their notes obtained from medical records following their discharge.

**Qualitative data collection**

Following the careful development of an appropriate semi-structured topic guide (see appendix), a sample of clinicians of all grades will have semi-structured interviews concerning their feelings and experience of DNACPR forms and the UFTO. These interviews may last up to an hour, although most will be significantly shorter, and be recorded, anonymised and transcribed prior to analysis. Clinicians will be drawn primarily from the wards where the UFTO is being trialled, but in addition, clinicians who come onto the wards where the UFTO is being used (for example outreach team nurses) will be approached for consent and interview. Open questions on difficulties, benefits, problems encountered, and feelings related to the decision-making process or discussions with patients will be asked, to facilitate exploration of the ethical implications of the process.

Observation of day-to-day practice of healthcare professionals will take place on all clinical areas directly involved in the research, in order to examine the role of

DNACPR/UFTO in hospital communications and behaviour.

**QUANTITATIVE ANALYSIS**

**Primary Outcome**

The primary endpoint of this trial is chosen to reflect the main study question and is both objective and measurable:

Appropriate (as defined by MEWS Score) and timely ( within 4 hours) referral to Outreach services or doctor:

The "Outreach team" are a team of nurses with acute care experience supported by Consultant Intensivists. They support ward staff looking after critically ill patients. The modified early warning system (MEWS) is a physiological scoring system that identifies patients at risk of deterioration who require increased levels of care [23] ( see below). Nurses are instructed to contact the Outreach Services or a doctor if the MEWS score of a patient reaches 4.


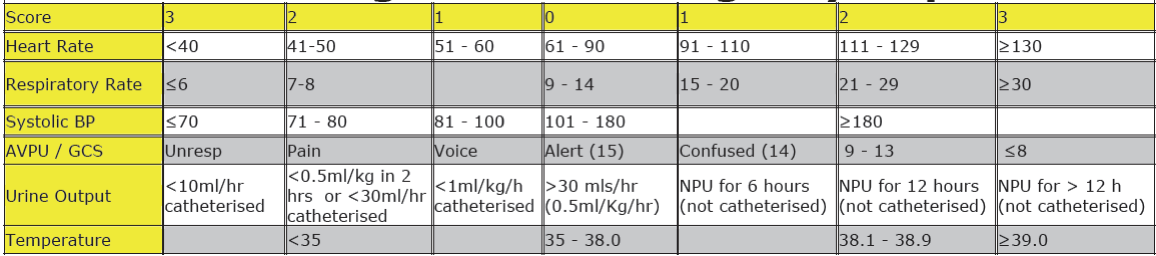


We will assess whether introduction of the UFTO alters frequency of appropriate referral (when MEWS ≥ to 4) to the Outreach team or to a doctor in those patients who have a not for attempted resuscitation instruction on their UFTO in comparison with those patients with conventional DNACPR forms. Referral will be defined as a doctor or the outreach seeing the patient within 4 hours of the MEWS being recorded as high.

All patients fall into one of the following categories (see flow chart, annex 4)

A. Observations not documented

B. MEWS ≤ 3 throughout hospital stay

C. MEWS ≥4, NOT referred to outreach or doctor

D. MEWS ≥4, referred to outreach or doctor

E. Observations documented, but MEWS not calculated by nursing staff. MEWS calculated by research team to be ≤ 3

F. Observations documented, but MEWS not calculated by nursing staff. MEWS calculated by research team to be ≥4

The case notes for the duration of a patient’s admission will be examined.

Patients in categories B, D and E are deemed to have been appropriately managed.

Patients in categories A, C and F are deemed to have been inappropriately managed.

Our primary endpoint is the number of patients who fall into categories A C or F. Of particular interest is the subgroup of patients with do not resuscitate instructions on them, in both the DNACPR and the UFTO arms.

**Secondary Outcomes**

The following will also be analysed and considered statistically significant at more stringent level 0.01 due to multiplicity of outcomes:

1. Assess whether introduction of the UFTO alters in isolation and as a composite endpoint frequency of referral to ICU,HDU,Medical Specialities, and out-of-hours medical care in those patients who have a not for attempted resuscitation instruction on their UFTO in comparison with those patients with conventional DNACPR forms, independent of MEWS scoring.

2. Assess whether the UFTO improves end of life care

3. Patient length of stay and in hospital mortality will also be examined

Recruitment rates will be monitored on an ongoing basis. No formal interim analyses are planned for this study. Confidence intervals and exact 2-sided p-values will be reported between group differences in outcomes and intention-to-treat analyses will be performed for whole group comparisons. The primary endpoint is restricted to the pre-defined subgroup of those with 'not for resuscitation' orders as described. Multivariate analyses using logistic regression for categorical endpoints, or multiple regression for continuous endpoints, will be used to develop suitable statistical models. Analyses will be carried out using SPSS software.

**QUALITATIVE ANALYSIS**

The qualitative data will be essential to investigate the contextual meanings that are attributed to the new form, and compare the extent to which it calls a different set of values and judgements to be made in comparison with the DNACPR form.

Anonymised interview recordings will be transcribed and entered into computer assisted qualitative data analysis software (Nvivo) for analysis. Data will remain anonymised and encrypted at all times. Key emergent themes using an abductive coding approach will establish the central issues raised by each group of respondents. Initial double blind coding of a sample of transcripts by the project team will generate a general coding frame and ensure inter-rater reliability. An open, iterative process will allow further development, and the consolidation of codes into a hierarchy of themes. Further, we will seek to establish whether explicit ethical issues are raised.

Thematic and content analysis of transcripts will reveal the extent to which the decision regarding resuscitation is contextualised within the broader context of treatment or whether it is conceptualised as a discrete issue. Transcripts will be searched for keywords (e.g. ‘duty’, ‘problem’, ‘dilemma’, ‘best interests’) associated with ethical problems, and the context of these words then examined for underlying ethical concepts.

The Nvivo database will be directly associated with the quantitative data in SPSS for further consolidation using basic descriptive statistics.

**Direct access to source data / documents**

We will, of course, permit trial related monitoring, audits, REC review, and regulatory inspections.

Ethical considerations

Consent

All patients will freely give their informed consent to participate in the study. A patient may decide to withdraw from the study at any time without prejudice to their future care.

Ethical committee review

The study protocol is to be seen and approved by the appropriate ethical review committee(s) of any participating hospital. Copies of the letters of approval are to be filed in the study file.

Declaration of Helsinki and ICH Good Clinical Practice

The study is to be carried out in conformation with the spirit and the letter of the declaration of Helsinki, and in accord with the ICH Good Clinical Practice Guidelines.

Data handling and record keeping

On the original dataset, we will number each of the patients in such a way that their personal details are not identifiable. This study number will then be used on all further analysis. Appropriate security and encryption will be supported by local data protection services.

Manual data will be in secured filing cabinets, in secure clinical areas.

Drs Fritz, Fuld, and Laroche along with the research nurse Alex Malyon and research assistant Jude Frankau will have access to personal data in the form of medical notes.

Quantitative data will be analysed primarily by Zoe Fritz in collaboration with Chris Palmer at the University of Cambridge Centre for Applied Medical Statistics. Qualitative data will be analysed primarily by Jude Frankau and Simon Cohn.

All personal data will be kept within the hospital grounds. Further analysis of anonymised data will be confined to the UK.

Financial and Insurance

We have been awarded a grant from the RfPB (NIHR) to carry out the research. The financial aspects will be run by Evgeny Dmitriev, the R&D Divisional Head of Finance at Addenbrooke’s Hospital. As the study is sponsored by the Cambridge University Hospital NHS Trust, insurance will be provided by the NHS Indemnity Scheme.

Dissemination and Publications policy

Accrual data will be published on the UKCRN database. The study is already ISRCTN registered and thus we are on the WHO international platform. We will circulate reports within the hospital, and publish on the intranet. We will submit our findings to peer review for publication; authorship will be shared by all of those who both contributed to the research and the manuscript preparation.

Supplements

Please see attached list of references, attached UFTO form, patient leaflet about discussing treatments and semi-structured interview topic guide.

Bibliography

1. National office of Statistics Series DHn. 68% of the population dies in hospital.

2. Aune S, Herlitz J, Bang A. Characteristics of patients who die in hospital with no attempt at resuscitation. Resuscitation. 2005 Jun;65(3):291-9.

3. Aronsky D, Kasworm E, Jacobson JA, Haug PJ, Dean NC, Aronsky D, et al. Electronic screening of dictated reports to identify patients with do-not-resuscitate status. J Am Med Inform Assoc. 2004 20040908;11(1067-5027 (Print)).

4. Decisions relating to cardiopulmonary resuscitation. A joint statement from the British Medical Association, the Resuscitation Council (UK) and the Royal College of Nursing. 2007.

5. Fritz Z, Fuld J. Ethical issues surrounding do not attempt resuscitation orders: decisions, discussions and deleterious effects. J Med Ethics. 2010 Oct;36(10):593-7.

6. Golin CE, Wenger NS, Liu H, Dawson NV, Teno JM, Desbiens NA, et al. A prospective study of patient-physician communication about resuscitation. Journal of the American Geriatrics Society. 2000;48(5 Suppl):S52-60.

7. Myint PK, Miles S, Halliday DA, Bowker LK. Experiences and views of specialist registrars in geriatric medicine on 'do not attempt resuscitation' decisions: a sea of uncertainty? QJM. 2006 Oct;99(10):691-700.

8. Sulmasy DP, Sood JR, Ury WA. Physicians' confidence in discussing do not resuscitate orders with patients and surrogates. J Med Ethics. 2008 20080131;34(1473-4257 (Electronic)).

9. Gorton AJ, Jayanthi NV, Lepping P, Scriven MW. Patients' attitudes towards "do not attempt resuscitation" status. J Med Ethics. 2008 Aug;34(8):624-6.

10. Stolman CJ, Gregory JJ, Dunn D, Ripley B. Evaluation of the do not resuscitate orders at a community hospital. Arch Intern Med. 1989 19890911;149(0003-9926 (Print)).

11. Cauchi L, Vigus J, Diggory P. Implementation of cardiopulmonary resuscitation guidelines in elderly care departments across: a survey of 13 hospitals shows wide variability in practice. Resuscitation. 2004 Nov;63(2):157-60.

12. La Puma J, Silverstein MD, Stocking CB, Roland D, Siegler M. Life-sustaining treatment. A prospective study of patients with DNR orders in a teaching hospital. Archives of Internal Medicine. 1988;148(10):2193-8.

13. Smith CB, Bunch O'Neill L. Do not resuscitate does not mean do not treat: how palliative care and other modalities can help facilitate communication about goals of care in advanced illness. Mount Sinai Journal of Medicine. 2008;75(5):460-5.

14. Uhlmann RF, Cassel CK, McDonald WJ. Some treatment-withholding implications of no-code orders in an academic hospital. Crit Care Med. 1984 Oct;12(10):879-81.

15. Fritz Z, Fuld J, Haydock S, Palmer C. Interpretation and intent: a study of the (mis)understanding of DNAR orders in a teaching hospital. Resuscitation. 2010 Sep;81(9):1138-41.

16. Beach MC, Morrison RS. The effect of do-not-resuscitate orders on physician decision-making. J Am Geriatr Soc. 2002 Dec;50(12):2057-61.

17. Chen JL, Sosnov J, Lessard D, Goldberg RJ. Impact of do-not-resuscitation orders on quality of care performance measures in patients hospitalized with acute heart failure. Am Heart J. 2008 Jul;156(1):78-84.

18. Cohen RI, Lisker GN, Eichorn A, Multz AS, Silver A. The impact of do-not-resuscitate order on triage decisions to a medical intensive care unit. J Crit Care. 2009 Jun;24(2):311-5.

19. Shepardson LB, Youngner SJ, Speroff T, Rosenthal GE. Increased risk of death in patients with do-not-resuscitate orders. Med Care. 1999 Aug;37(8):727-37.

20. Wenger NS, Pearson ML, Desmond KA, Brook RH, Kahn KL. Outcomes of patients with do-not-resuscitate orders. Toward an understanding of what do-not-resuscitate orders mean and how they affect patients. Archives of Internal Medicine. 1995;155(19):2063-8.

21. Mittelberger JA, Lo B, Martin D, Uhlmann RF. Impact of a procedure-specific do not resuscitate order form on documentation of do not resuscitate orders. Arch Intern Med. 1993 Jan 25;153(2):228-32.

22. O'Toole EE, Youngner SJ, Juknialis BW, Daly B, Bartlett ET, Landefeld CS. Evaluation of a treatment limitation policy with a specific treatment-limiting order page. Arch Intern Med. 1994 Feb 28;154(4):425-32.

23. Davila F, Boisaubin EV, Sears DA. Patient care categories: an approach to do-not-resuscitate decisions in a public teaching hospital. Crit Care Med. 1986 19870106;14(0090-3493 (Print)).

24. Tolle SW, Tilden VP, Nelson CA, Dunn PM. A prospective study of the efficacy of the physician order form for life-sustaining treatment. J Am Geriatr Soc. 1998 Sep;46(9):1097-102.
