# Supplementary material for: The Universal Form of Treatment Options (UFTO) as an Alternative to Do Not Attempt Cardiopulmonary Resuscitation (DNACPR) Orders: A Mixed Methods Evaluation of the Effects on Clinical Practice and Patient Care
Source: PLoS One. 2013 Sep 4;8(9):e70977. doi: 10.1371/journal.pone.0070977 (PMC3762818; doi:10.1371/journal.pone.0070977)
Supplement: Appendix S1 — Online appendix including details of Standard Operating Procedures, protocol amendments, Patient Information leaflet “Talking with your doctor” and detailed breakdown of quantitative and qualitative data. (DOCX) [file pone.0070977.s003.docx]

# Appendix S1. Online appendix

# Title page

Supplementary Appendix for “The Universal Form of Treatment Options (UFTO) as an Alternative to Do Not Attempt Cardiopulmonary Resuscitation (DNACPR) Orders: A Mixed Methods Evaluation of the Effects on Clinical Practice and Patient Care”

# Table of Contents

[Title page 1](#_Toc359322794)

[Table of Contents 2](#_Toc359322795)

[List of Investigators 5](#_Toc359322796)

[Statistical Method 6](#_Toc359322797)

[Sample size. 6](#_Toc359322798)

[Endpoint of Global Trigger Tool Assessed ‘harm events’ 6](#_Toc359322799)

[Qualitative Methodology, Methods, and Data Generation 8](#_Toc359322800)

[Qualitative Data Analysis 9](#_Toc359322801)

[Results 10](#_Toc359322802)

[Comparison of patients ‘Not for Resuscitation’ on the study wards in the DNACPR and UFTO periods 10](#_Toc359322803)

[Rate of harms per 100 patient admissions 10](#_Toc359322804)

[Rate of harms per 1000 patient-days 10](#_Toc359322805)

[Comparison of patient characteristics 11](#_Toc359322806)

[Multivariate regression 11](#_Toc359322807)

[Sensitivity analysis – including palliative patients 12](#_Toc359322808)

[Rate of harms per 1000 patient-days 12](#_Toc359322809)

[Comparison of patient characteristics 13](#_Toc359322810)

[Multivariate regression 13](#_Toc359322811)

[Severity of harms 14](#_Toc359322812)

[Preventability of harms 14](#_Toc359322813)

[Contemporaneous controls- patients not for resuscitation on wards not participating in study 14](#_Toc359322814)

[Rate of harms per 100 patient admissions 14](#_Toc359322815)

[Rate of harms per 1000 patient-days 15](#_Toc359322816)

[Comparison of patient characteristics 15](#_Toc359322817)

[Multivariate regression 15](#_Toc359322818)

[Contemporaneous controls – patients for resuscitation on wards participating in the study 16](#_Toc359322819)

[Rate of harms per 100 patient admissions 16](#_Toc359322820)

[Rate of harms per 1000 patient-days 16](#_Toc359322821)

[Distribution of harms in each ward 17](#_Toc359322822)

[Comparison of patient characteristics 17](#_Toc359322823)

[Distribution of excluded patients 17](#_Toc359322824)

[Mortality breakdown 18](#_Toc359322825)

[Qualitative results 18](#_Toc359322826)

[Standard Operating Procedure for completing a case note review using the IHI Global Trigger Tool 19](#_Toc359322827)

[Figures 29](#_Toc359322828)

[Figure S1: Plot of subgroup means against variances 29](#_Toc359322829)

[Figure S2: Bar chart showing frequency of each type of harm for trigger categories within UFTO and DNACPR groups 30](#_Toc359322830)

[Figure S3: Bar chart showing within-group percentages of harms foreach category of severity (NCC MERP Index). 31](#_Toc359322831)

[Figure S4: Bar chart showing within-group percentages of harms for each category of preventability 31](#_Toc359322832)

[Figure S5 “Talking with your doctor about treatments: a guide for patients” 32](#_Toc359322833)

[Figure S6: the Global Trigger Tool, UK version 35](#_Toc359322834)

[**Figure S7: Illustration of adapted Framework Analysis of interview data** 36](#_Toc359322835)

[Figure S8: Simplified illustration of how thematic framework progressed 37](#_Toc359322836)

[Tables 38](#_Toc359322837)

[Rates of harm in patients ‘Not for Resuscitation’ in the UFTO and DNACPR periods 38](#_Toc359322838)

[Table S1: The average number of harms per 100 patient admissions, stratified by ward. 38](#_Toc359322839)

[Table S2: The average number of harms per 1000 patient-days, stratified by ward. 38](#_Toc359322840)

[Table S3: Comparison of patient characteristics between groups. 38](#_Toc359322841)

[Table S4: Regression coefficients for group (UFTO or DNACPR) expressed as per 100 patient admissions 39](#_Toc359322842)

[Rates of harms for patients ‘Not for Resuscitation’ in the DNACPR and UFTO period- including palliative care patients 39](#_Toc359322843)

[Table S5: The average number of harms per 100 patient admissions, stratified by ward. 39](#_Toc359322844)

[Table S6: The average number of harms per 1000 patient-days, stratified by ward. 40](#_Toc359322845)

[Table S7: Comparison of patient characteristics between groups 40](#_Toc359322846)

[Table S8: Regression coefficients for group (UFTO or DNACPR) expressed as per 100 patient admissions. 40](#_Toc359322847)

[Table S9: The frequency of each type of harm for trigger categories within UFTO and DNACPR groups 41](#_Toc359322848)

[Table S10: Rating of severity of harms using NCC MERP Index severity of the harms in DNACPR and UFTO groups 43](#_Toc359322849)

[Table S11: severity of harms collapsed in preparation for a Chi-squared test for trend (with all expected cell values more than 4) 43](#_Toc359322850)

[Table S12: The distribution of preventability of harms in each group. 43](#_Toc359322851)

[Table S13: preventability of harms collapsed in preparation for a Fisher’s Exact test 44](#_Toc359322852)

[Contemporaneous controls- patients not for resuscitation on wards not participating in study 44](#_Toc359322853)

[Table S14: Comparison of patient characteristics between groups for patients on non study wards during the DNACPR period (May-July 2010) and UFTO period (Dec 2010-Jan 2011) 44](#_Toc359322854)

[Table S15a: Comparison between characteristics of patients not for resuscitation on study wards and on control wards during the DNACPR period. 45](#_Toc359322855)

[Table S16: Regression coefficients for group (UFTO or DNACPR) on control wards expressed as per 100 patient admissions. 46](#_Toc359322856)

[Table S17: Comparison of patient characteristics between groups (UFTO or DNACPR periods) for patients remaining for resuscitation on study wards. 46](#_Toc359322857)

[Table S18: Exclusions from dataset on study wards during DNACPR and UFTO periods. 47](#_Toc359322858)

[Mortality Tables 47](#_Toc359322859)

[Table S19: 30 day mortality in those patient not for resuscitation 47](#_Toc359322860)

[Table S20:30 day mortality in palliative care patients 47](#_Toc359322861)

[Table S21:30 day mortality for other excluded patients 47](#_Toc359322862)

[Table S22: 30 day mortality in those patients for resuscitation on the study wards 48](#_Toc359322863)

[Table S23: Total 30 day mortality 48](#_Toc359322864)

[Qualitative Tables 48](#_Toc359322865)

[Table S24: Breakdown of Interview Participants by Clinical Grade 48](#_Toc359322866)

[Table S25: Identifying a thematic framework: Level 1 and 2 coding for whole data set 48](#_Toc359322867)

[Table S26: Indexing: example of full descriptive coding labels under level 1 code 'UFTO' 49](#_Toc359322868)

[Table S27: Example of charting: the theme of ‘Timing’ 52](#_Toc359322869)

[Table S28 Stage 5 - Mapping and Interpretation: Key Themes emergent from Interview data 54](#_Toc359322870)

# List of Investigators

Zoë Fritz, Cambridge University Hospitals NHS Foundation Trust, UK

Alexandra Malyon, Cambridge University Hospitals NHS Foundation Trust, UK

Jude Frankau, Cambridge University Hospitals NHS Foundation Trust, UK

Richard Parker, Department of Public Health and Primary Care, Institute of Public Health, Cambridge University

Simon Cohn, Department of Public Health and Primary Care, Institute of Public Health, Cambridge University

Chris Palmer, Department of Public Health and Primary Care, Institute of Public Health, Cambridge University

Clare Laroche, West Suffolk Hospital, Bury St Edmunds

Jonathan Fuld, Department of Public Health and Primary Care, Institute of Public Health, Cambridge University

# Statistical Method

## Sample size.

A before and after study was planned using timely referrals of patients with an Early Warning Score (EWS) of greater than 3 as a primary endpoint. The power calculation was constructed assuming a two-sided Fisher's exact test will be performed at the 5% significance level, on a EWS outcome representing the proportion of patients inappropriately managed. A sample size of 108 individuals with 'Not for Resuscitation' orders per group was thought to provide 80% power to detect an absolute difference of 20% between the UFTO and DNACPR groups in the proportion of patients inappropriately managed (as defined by the EWS). It was anticipated, using preliminary data, that this number of patients would be admitted onto the study wards in a 3 month period.

The frequency of EWS greater than 3 did not occur at the same rate as in the preliminary data, and there was no statistical significance in these results. ethics approval was therefore obtained for a further data set to be collected to assess the Global Trigger Tool in patients in these predefined periods. (The GTT was not in use in the UK when the initial study was planned). A higher statistical stringency of 2% was required for significance for this outcome.

## Endpoint of Global Trigger Tool Assessed ‘harm events’

The secondary endpoint was the rate of harms per 1000 patient bed days in those patients “not for resuscitation”, calculated separately for the group of patients with a DNACPR order and those receiving UFTO. The rate of harms per 100 patient admissions was also calculated within each patient group. The UFTO and DNACPR groups were compared by calculating the absolute rate difference between the groups with a corresponding 95% confidence interval. A stratified analysis was then performed by calculating the rate differences within each ward group (Wards A & B). Patient characteristics were compared between groups using the Fisher’s Exact test for all categorical variables and the Mann-Whitney test for all continuous variables except age, for which an independent samples t-test was used. This was because all continuous variables except age had non-normal distributions.

The frequency distribution of the type of harms, and the severity and preventability of harms was tabulated for each group. The chi-squared test for trend was utilized where possible to statistically compare between the groups. However, in the case of tables with low expected counts, Fisher’s Exact tests were used.

Difference in proportions were calculated to compare the number of harms contributing to patient death and the number of harms preventable at any level between groups, with corresponding 95% confidence intervals calculated using a recommended method (Newcombe’s method) [1]. The same method was used to compare discussion rates, EWS response and mortality between groups.

A Poisson regression model was fitted to the number of harms data to evaluate the effect of group (UFTO/NFR or DNACPR) on number of harms after adjusting for possible confounders. For the first model, only the group variable was included as an explanatory variable in the model; and then ward was also added; and finally, a model was fitted including all covariates of interest: ward, gender, Charlson co-morbidity score, MEWS score and age at admission. For all models, a log-transformed offset term was included for hospital length of stay, to adjust for differences in periods of observation across patients.

As a sensitivity analysis, negative-binomial regression models were also fitted to account for any over-dispersion in the data. As before, a log-transformed offset term was included for hospital length of stay, to adjust for differences in periods of observation across patients.

To determine the effect of including palliative care patients in the analysis, exactly the same statistical methods were employed as above but with the addition of the palliative care patients.

Additional assessments, using the same analysis as outlined above, were carried out on two contemporaneous case control groups:

1. Those for resuscitation on the wards studied during the same time period,
2. Those not for resuscitation on two wards where the intervention (the Universal Form of Treatment Options) was not introduced, during the same time periods.

R software version 2.15.0 [2] was used for most analyses, except SPSS software version 18 [3] was used for data manipulation and to calculate summary statistics and p-values when comparing patient characteristics between groups.

[1] Altman, D.G., Machin, D., Bryant, T.N., and Gardner, M.J. (2000). Statistics with Confidence: Second Edition, pages 45-55 (Chapter 6), BMJ books.

[2] R Development Core Team (2012). R: A language and environment for statistical computing. R Foundation for Statistical Computing, Vienna, Austria. ISBN 3-900051-07-0, URL <http://www.R-project.org/>.

[3] **SPSS/PASW for Windows, Rel. 18.0.3. 2010. Chicago: SPSS Inc.**

# Qualitative Methodology, Methods, and Data Generation

The methodological orientation underpinning the qualitative research was that of ethnography. The researcher undertaking this work (JMF), as a postgraduate-qualified social anthropologist with previous experience in health services research was employed as a research assistant within the project. She had no previous relationship established with the hospital or the wards where the research took place prior to the commencement of the study and was not clinically qualified. Senior members of the clinical team where the research took place were aware that she was part of a research team looking at an alternative to the DNACPR form from the beginning of the study. Other members of clinical staff knew in the first phase of the study (before UFTO education) that she was researching current DNACPR practice and in the second phase that she was researching use of the UFTO. On the wards, during data generation JMF was positioned as a non-clinician member of staff – with access to the majority of areas of the ward (including staff-only areas), allowed to shadow clinical staff in a variety of their duties and observe ward practice as long as she did not obstruct it in any way. In this role, staff on the wards came to accept and ignore her as part of the ‘scenery’ of the ward, occasionally asking questions or requesting interviews but for the most part quietly in the background observing and making field-notes. Observation was undertaken on the two wards on a daily basis for the duration of the study, with JMF also taking part in the UFTO education phase as an educator.

Participants for interview were purposively sampled in order to gather views from a range of clinical grades. In the first instance, participants were invited for interview by e-mail and this was followed up by face-to-face requests. No-one who was approached for interview declined to participate. Informed consent was gained from all interviewees. Interviews lasted between 10 minutes and one hour, based on time restraints due to clinical duties (on the whole, the more senior the clinician grade, the more time they felt able to spend being interviewed). No-one besides the interviewer and the participant was present during interviews, except for the group interview of outreach nurses where four nurses and the interviewer were present. A semi-structured interview guide was used by the interviewer, allowing interviewees to shape the interview following themes which were important for them about the topics discussed. All interviews were audio-recorded, transcribed verbatim and uploaded into Nvivo 8.0 to facilitate data analysis.

# Qualitative Data Analysis

For the qualitative data analysis, we used a framework analysis, adapted from Ritchie and Spencer (1994). They describe 5 key stages for framework analysis of qualitative data as:

1. Familiarisation
2. Identifying a thematic framework
3. Indexing
4. Charting
5. Mapping and Interpretation

- Richie, J and Spencer, L (1994), ‘Qualitative data analysis for applied policy research’, in Bryman and Burgess, eds., Analysing *Qualitative Data,* London: Routledge, p173-194.

We adapted their approach to framework analysis to include sub-stages of descriptive coding within the indexing stage and refinement of themes in order to allow for the identification and inclusion of emergent themes as well as *a priori* ones. Data generated was analysed by JMF and a senior medical anthropologist [SC]. The qualitative data were discussed with physician colleagues [ZF & JF], during which their clinical experience helped to further contextualise the interpretation of results.

# Results

## Comparison of patients ‘Not for Resuscitation’ on the study wards in the DNACPR and UFTO periods

### Rate of harms per 100 patient admissions

There were 44 harms among 118 patients in the UFTO group (admissions Nov. 2010-Jan 2011). This equates to an estimated 37.3 harms per 100 patient admissions with an approximate 95% confidence interval of 27.7 to 50.1 harms per 100 patient admissions.

In comparison, there were 71 harms among 103 patients in the DNACPR group (admissions May-July 2010). This equates to an estimated 68.9 harms per 100 patient admissions with an approximate 95% confidence interval of 54.6 to 87.0 per 100 patient admissions.

The rate difference per 100 patient admissions (DNACPR – UFTO) was calculated to be 31.6 harms with a 95% confidence interval of 12.2 to 51.1 (p-value 0.001). Therefore, the rate of harms per 100 patient admissions was significantly higher in the DNACPR group compared to the UFTO group.

Table S1 shows the average number of harms per 100 patient admissions, stratified by ward. Approximate 95% confidence intervals are shown in brackets.

Within each ward stratum, the DNACPR group had a significantly higher rate of harms per 100 patient admissions than the UFTO group.

For patients in the ward A, the DNACPR group had a significantly higher rate of harms per 1000 patient-days than the UFTO group. However, for patients in ward B there was insufficient evidence that the rate of harms was higher in the DNACPR group than the UFTO group.

### Rate of harms per 1000 patient-days

There were 44 harms in the UFTO group with a total of 2021 patient-days at risk of harm in hospital. There were 71 harms in the DNACPR group with a total of 2048 patient-days at risk of harm in hospital.

Therefore, the rate of harm in the UFTO group was calculated to be 21.8 per 1000 patient-days and the rate of harm in the DNACPR group was calculated to be 34.7 per 1000 patient days.

The rate difference in harms (DNACPR – UFTO) was 12.9 per 1000 patient-days with a 95% confidence interval of 2.6 to 23.2 (p-value 0.01). Therefore, there was a significant difference in the rate of harms between the groups. Patients in the DNACPR group had a significantly greater rate of harm compared to the UFTO group.

### Comparison of patient characteristics

Patient characteristics appear to be very similar between the groups. There were no significant differences at the 5% level (Table S3).

### Multivariate regression

A Poisson regression model was fitted to the number of harms data to evaluate the effect of group (UFTO or DNACPR) on number of harms after adjusting for possible confounders. For the first model, only the group variable was included as an explanatory variable in the model; and then ward was also added; and finally, a model was fitted including all covariates of interest: ward, gender, Charlson comorbidity score, MEWS score and age at admission. For all models, a log-transformed offset term was included for hospital length of stay, to adjust for differences in periods of observation across patients. The results are shown in the table S4.

The Poisson regression model relies on the assumption of a 1:1 mean variance relationship; such that the mean is assumed to be equal to the variance among all patient subgroups. A graph of subgroup means against variances indicates the possibility of a slightly curved relationship between the mean and variance, although it is not very obvious (Figure S1).

As a sensitivity analysis, we fitted negative-binomial regression models which account for any over-dispersion in the data. As before, a log-transformed offset term was included for hospital length of stay, to adjust for differences in periods of observation across patients.

It should be noted that although coefficients are expressed as per 100 patient admissions, length of hospital stay is taken into account in the analysis. Corresponding 95% confidence intervals and p-values are also presented. Akaike’s Information Criterion (AIC) is also included to enable comparison between models.

AICs are very similar between models, but the simple Poisson regression model with group as covariate appears to be the most parsimonious model (i.e. has the lowest AIC).

The models show a significant difference in rate of harm between the groups at the 5% level; even after adjusting for ward, age, gender, MEWS score and Charlson comorbidity score. This suggests that patients in the DNACPR group have a greater rate of harm compared to those in the UFTO group, and confirms our earlier conclusion.

## Sensitivity analysis – including palliative patients

*Rate of harms per 100 patient admissions*

There were 47 harms among 138 patients in the UFTO group. This equates to an estimated 34.1 harms per 100 patient admissions.

In comparison, there were 72 harms among 108 patients in the DNACPR group. This equates to an estimated 66.7 harms per 100 patient admissions.

The rate difference per 100 patient admissions (DNACPR – UFTO) was calculated to be 32.6 harms with a 95% confidence interval of 14.4 to 50.8 (p-value 0.0005). Therefore, the rate of harms per 100 patient admissions was significantly higher in the DNACPR group compared to the UFTO group.

Table S5 shows the average number of harms per 100 patient admissions, stratified by ward. Approximate 95% confidence intervals are shown in brackets.

Therefore, within each ward stratum, the DNACPR group had a significantly higher rate of harms per 100 patient admissions than the UFTO group.

### Rate of harms per 1000 patient-days

There were 47 harms in the UFTO group with a total of 2413 patient-days at risk of harm in hospital. There were 72 harms in the DNACPR group with a total of 2106 patient-days at risk of harm in hospital.

Therefore, the rate of harm in the UFTO group was calculated to be 19.5 per 1000 patient-days and the rate of harm in the DNACPR group was calculated to be 34.2 per 1000 patient days.

The rate difference in harms (DNACPR – UFTO) was 14.7 per 1000 patient-days with a 95% confidence interval of 5.0 to 24.4 (p-value 0.003). Therefore, there was a significant difference in the rate of harms between the groups. Patients in the DNACPR group had a significantly greater rate of harm compared to the UFTO group.

Table S6 shows the average number of harms per 1000 patient-days, stratified by ward. Approximate 95% confidence intervals are shown in brackets.

For patients within each ward, the DNACPR group had a significantly higher rate of harms per 1000 patient-days than the UFTO group.

### Comparison of patient characteristics

Table S7 shows a comparison of patient characteristics between groups for the sensitivity analysis.

Patient characteristics appear to be very similar between the groups. There were no significant differences at the 5% level.

### Multivariate regression

A Poisson regression model was fitted to the number of harms data to evaluate the effect of group (UFTO or DNACPR) on number of harms after adjusting for possible confounders. For the first model, only the group variable was included as an explanatory variable in the model; and then ward was also added; and finally, a model was fitted including all covariates of interest: ward, gender, Charlson comorbidity score, MEWS score and age at admission. For all models, a log-transformed offset term was included for hospital length of stay, to adjust for differences in periods of observation across patients. The results are shown in the table S8.

We also fitted negative-binomial regression models which account for any over-dispersion in the data. As before, a log-transformed offset term was included for hospital length of stay, to adjust for differences in periods of observation across patients.

Table S8 also shows the regression coefficients for group (UFTO or DNACPR) expressed as per 100 patient admissions. Please note that although coefficients are expressed as per 100 patient admissions, length of hospital stay is taken into account in the analysis. Corresponding 95% confidence intervals and p-values are also presented. Akaike’s Information Criterion (AIC) is also included to enable comparison between models.

AICs are very similar between models, but the Poisson regression model with group and ward as covariates appears to be the most parsimonious model (i.e. has the lowest AIC).

**The models show a significant difference in rate of harm between the groups at the 5% level; even after adjusting for ward, age, gender, MEWS score and Charlson comorbidity score. This suggests that patients in the DNACPR group have a greater rate of harm compared to those in the UFTO group, and confirms our earlier conclusion..**

Table S9 shows the frequency of each type of harm within each group. The harms are listed in order according to the frequencies in the DNACPR group. Please note that the numbers are small; so although percentages are given to aid comparisons between groups, they are unlikely to be reliable estimates of the true distribution of harms in the general population. A bar chart showing the absolute number of patients with each type of harm is shown in Figure S2.

### Severity of harms

Table S10 shows the distribution of severity of harms in each group. Figure S3 shows the percentage of harms within each category of severity.

Table S10 was collapsed in preparation for a Chi-squared test for trend (with all expected cell values more than 4). This is displayed in Table S11.

Applying a Chi-squared test for trend to Table S11 produces a chi-squared test statistic of 6.34 with a p-value of 0.01. Therefore, the severity of harms is significantly higher in the DNACPR group compared to the UFTO group.

### Preventability of harms

Table S12 below shows the distribution of preventability of harms in each group. (Fisher’s Exact test p-value 0.67).

Figure S4 shows the distribution of harms according to their preventability.

Table S12 was collapsed to ensure that all cells have expected values of at least 4 (Table S13).

Applying a Fisher’s Exact test to Table S13, we obtain a p-value of 0.70, which is non-significant at the 5% level. Therefore, there is insufficient evidence of a difference in the preventability of harms between the groups.

## Contemporaneous controls- patients not for resuscitation on wards not participating in study

### Rate of harms per 100 patient admissions

There were 17 harms among 25 patients in the UFTO period. This equates to an estimated 68 harms per 100 patient admissions with an approximate 95% confidence interval of 42 to 109 harms per 100 patient admissions.

In comparison, there were 13 harms among 25 patients in the DNACPR period. This equates to an estimated 52 harms per 100 patient admissions with an approximate 95% confidence interval of 30 to 90 per 100 patient admissions.

The rate difference per 100 patient admissions (UFTO period – DNACPR period) was calculated to be 16 harms with a 95% confidence interval of -26.9 to 58.9 (p-value 0.47). Therefore, the rate of harms per 100 patient admissions was not significantly different between the UFTO and DNACPR periods.

### Rate of harms per 1000 patient-days

There were 17 harms in the UFTO period with a total of 527 patient-days at risk of harm in hospital. There were 13 harms in the DNACPR period with a total of 718 patient-days at risk of harm in hospital.

Therefore, the rate of harm in the UFTO period was calculated to be 32 per 1000 patient-days and the rate of harm in the DNACPR period was calculated to be 18 per 1000 patient days.

The rate difference in harms (UFTO – DNACPR period) was 14.2 per 1000 patient-days with a 95% confidence interval of -4.1 to 32.4 (p-value 0.13). Therefore, there was no significant difference in the rate of harms between the groups.

### Comparison of patient characteristics

Table S14 shows a comparison of patient characteristics between groups.

The median Charlson comorbidity score of patients was significantly higher in the UFTO period compared to the DNACPR period. The Mann-Whitney test gave a p-value of 0.04, which is significant at the 5% level.

### Multivariate regression

A Poisson regression model was fitted to the number of harms data to evaluate the effect of period (UFTO or DNACPR) on number of harms after adjusting for possible confounders. For the first model, only the group variable was included as an explanatory variable in the model; and then a model was fitted including all covariates of interest: ward, gender, Charlson comorbidity score, MEWS score and age at admission. For all models, a log-transformed offset term was included for hospital length of stay, to adjust for differences in periods of observation across patients. The results are shown in the Table S16.

As a sensitivity analysis, we fitted negative-binomial regression models which account for any over-dispersion in the data. As before, a log-transformed offset term was included for hospital length of stay, to adjust for differences in periods of observation across patients.

Table 16 shows the regression coefficients for group (UFTO or DNACPR) expressed as per 100 patient admissions. Corresponding 95% confidence intervals and p-values are also presented. Akaike’s Information Criterion (AIC) is also included to enable comparison between models.

AICs are very similar between models, but the negative binomial regression model with group as covariate appears to be the most parsimonious model (i.e. has the lowest AIC).

The models show insufficient evidence of a difference in rate of harm between the UFTO and DNACPR periods at the 5% level; even after adjusting for ward, age, gender, MEWS score and Charlson comorbidity score.

Table S15a and S15b shows a comparison of patient characteristics between patients on the study wards and those from the case control group on non-study wards for the DNACPR period and UFTO periods respectively. There were no significant differences between the study wards and non-study wards in the UFTO period. The patients on the study wards were also mostly similar to those on the non-study wards in the DNACPR period. However, the non-study ward patients had a significantly longer length of hospital stay compared to those on the study wards during the DNACPR period.

## Contemporaneous controls – patients for resuscitation on wards participating in the study

### Rate of harms per 100 patient admissions

There were 5 harms among 58 patients in the UFTO group. This equates to an estimated 8.6 harms per 100 patient admissions with an approximate 95% confidence interval of 3.6 to 20.7 harms per 100 patient admissions.

In comparison, there were 4 harms among 60 patients in the DNACPR group. This equates to an estimated 6.7 harms per 100 patient admissions with an approximate 95% confidence interval of 2.5 to 17.8 per 100 patient admissions.

The rate difference per 100 patient admissions (DNACPR - UFTO) was calculated to be -2.0 harms with a 95% confidence interval of -11.9 to 8.0 (p-value 0.70). Therefore, the rate of harms per 100 patient admissions was not significantly different between the UFTO and DNACPR groups.

### Rate of harms per 1000 patient-days

There were 5 harms in the UFTO group with a total of 685 patient-days at risk of harm in hospital. There were 4 harms in the DNACPR group with a total of 561 patient-days at risk of harm in hospital.

Therefore, the rate of harm in the UFTO group was calculated to be 7.3 per 1000 patient-days and the rate of harm in the DNACPR group was calculated to be 7.1 per 1000 patient days.

The rate difference in harms (DNACPR – UFTO) was -0.2 per 1000 patient-days with a 95% confidence interval of -9.6 to 9.3 (p-value 0.97). Therefore, there was no significant difference in the rate of harms between the groups.

### Distribution of harms in each ward

Within ward B, there were 3 harms among 28 patients (or 3 harms in 290 patient days) in the DNACPR group; compared to 2 harms among 24 patients (or 2 harms in 318 patient days) in the UFTO group.

Within ward A, there was 1 harm among 32 patients (or 1 harm in 271 patient days) in the DNACPR group; compared to 3 harms among 34 patients (or 3 harms in 367 patient days) in the UFTO group.

### Comparison of patient characteristics

Table S17 shows a comparison of patient characteristics in the control group, those remaining for resuscitation on the study wards during the two periods (UFTO-DNACPR)

Patient characteristics appear to be very similar between the groups. There were no significant differences at the 5% level.

### Distribution of excluded patients

Table S18 shows a comparison of patient exclusions on the study wards during the two periods (UFTO-DNACPR) . There were more patients identified as being for palliative care in the UFTO group; all palliative care patients were therefore subsequently reincluded in a further analysis

## Mortality breakdown

Mortality breakdown is given tables S19-23.

In the DNAR period those not for resuscitation and not for palliative care there were 45 deaths; the same group in the UFTO period there were 35 deaths. Of the palliative care patients there were 4 deaths in the DNAR period and 16 In the UFTO period. Since there was an increase in the total number of patients recognised as being for palliative care, we re-included these patients a further analysis. There were a further number of deaths within 30 days in patients who remained for resuscitation: 7 in the DNACPR group and 15 in the UFTO group. The increase in this group may reflect that the UFTO period was over the winter months (Nov2011 - Jan 2012) while the DNACPR period was over the summer (May-July 2011).

## Qualitative results

Please see tables S24-28 for details of Breakdown of Interview Participants by Clinical Grade; Level 1 and 2 coding for whole data set; examples of full descriptive coding labels; and Key Themes emergent from Interview data

# Standard Operating Procedure for completing a case note review using the IHI Global Trigger Tool

**A Universal Form of Treatment Options (UFTO): A study to evaluate the UFTO as an alternative to the DNACPR.**

UFTO Research Group Standard Operating Procedure

| SOP Number/ Version |  | Effective Date |
| --- | --- | --- |
| SOP 4 / V1.0 |  | Review Date |

| Author  Designation: | Signature | Date |
| --- | --- | --- |
| Approved by Manager:  Designation: |  |  |

**BACKGROUND**

The concept of using clues or ‘triggers’ to identify adverse events from a medical case note review has been widely used. Trigger tools were initially developed to help identify adverse medication events, with the methodology later being adapted to measure adverse events in areas such as Critical Care.

The Institute of Health Improvement (IHI) subsequently developed the IHI Global Trigger Tool for Measuring Adverse Events. The tool is designed to identify adverse events in adult inpatients throughout the hospital. A UK version has become widely used since 2009.

The Global Trigger Tool (GTT) provides a reliable and validated method of identifying adverse events and a way of tracking these rates of harm over time. Tracking adverse events is a useful way of assessing whether patient safety interventions are improving care.

The trigger tool methodology uses a random, retrospective review of patient medical records to identify ‘triggers’ such as a patient fall or an Early Warning Score (EWS) requiring action, which may be an indication that harm has occurred. Once a trigger is identified the patient medical record will be further reviewed to establish whether an adverse event occurred.

The IHI Global Trigger Tool defines an adverse event as ***any physical harm to the patient***. The tool limits the definition of adverse events to physical rather than emotional harm. If an adverse event is identified then a level of harm will be assigned to this harm.

**PURPOSE**

The purpose of this SOP is to describe the procedure and process for carrying out a review of the case notes of patients using the IHI Global Trigger Tool for the purpose of the UFTO research study.

**RESPONSIBILITIES**

This SOP will apply to all members of the study team responsible for carrying out case note reviews using the GTT.

All staff carrying out case note review using the GTT will be appropriately qualified physicians or experienced nurses. Reviewers will have completed the IHI online training and completed reviews using the online training records. A record of members of the review teams and the training they have undertaken will be kept.

**PROCEDURE**

1. **Review Teams**

The review teams for manual case note review will consist of two primary reviewers and two Consultant Physician reviewers.

The primary reviewers can be anyone who is knowledgeable about the case notes and care provided in the hospital. In this case the primary review team will consist of an experienced nurse and a junior doctor.

Two Consultant Physician reviewers will review all triggers at the second stage of review.

1. **Record review**

**Primary record review**

- Records will be reviewed in a random order.

- The review of the first 20 randomly selected records will be carried out by two separate reviewers to establish inter-reviewer reliability and ensure a standardisation of the process.

- Primary reviewers will then conduct a review of each record using the Global Trigger Tool (GTT). The 29 GTT modules being used in this case are detailed below and of the Tool included in Figure S6. Only the modules from the GTT that apply to the record being reviewed will be included. All reviews should include general care, medication and laboratory test modules. Other modules e.g. the Intensive care module will only be used if applicable to that admission.

-The primary record review is carried out in a standardised fashion in 20 minutes or less. The review is carried out in the following order:

- Discharge diagnosis
- Discharge summary
- Medication charts and the medication administration documentation
- Laboratory results
- Nursing documentation
- Physician case notes
- If time permits any other areas of the case notes.

- Reviewers will indentify potential triggers using the GTT.

- A record of any triggers identified will be inputted directly into an electronic ‘FileMaker’ database. No assessment will be made at this stage of association of the trigger with harm.

-If the primary reviewer identifies a trigger they will then prepare a short, one to two paragraph summary describing the event. This information will also be inputted directly into a field in the FileMaker database.

-This record will be linked to other study data previously collected from these patients’ case notes. The other data will be hidden from primary reviewers at the time of record review to reduce potential reviewer bias.

-Multiple triggers can be recorded for each patient. The criteria for identification of triggers are included later in this SOP.

-Primary reviewers review paper patient records, it is not possible for identifying information to be removed from these therefore primary reviews will not to be blinded.

**Secondary Review**

- The information on all triggers collected by primary reviewers will then be passed on to two Consultant Physician reviewers who will make final determination of presence, severity and preventability of harm.

-All other information relating to the patient and the admission will be concealed from physician reviewers to reduce bias. Secondary reviewers will not be aware of which study group the admission was from.

-The secondary reviewers will independently review the GTT triggers and make an assessment of harm. Their determination will then be compared to establish concordance. If there is any disagreement between the physician reviewers as to the assessment of harm, the cases will be discussed and consensus reached. Inter-rater reliability on determination of harm will be established from pre discussion ratings.

- The secondary reviewers will be responsible for making a final assessment on the presence of harm.

- If they assess that harm has occurred they will then assign a severity to the harm. This will be done using the index of the National Coordinating Council for Medication Error Reporting and Prevention (NCC MERP) to evaluate severity of harm. The tool does not include categories A-D as these describe errors that do not cause harm. The tool includes categories E, F, G, H, and I of the NCC MERP Index, because these categories describe errors that do cause harm. The categories of severity are:

- Category E: Temporary harm to the patient and required intervention
- Category F: Temporary harm to the patient and required initial or prolonged hospitalisation
- Category G: Permanent patient harm
- Category H: Intervention required to sustain life
- Category I: Patient death

- Secondary physician reviewers will then also make a determination about the preventability of the harm. A Likert scale will be used to evaluate preventability with 1 for “definitely not preventable” and 4 for “definitely preventable”.

- All data on presence, severity and preventability of harm will be inputted directly into the ‘FileMaker’ database.

3. **Triggers**

In order to maximise concordance and minimise the impact of confounding, certain criteria were established in relation to the GTT triggers prior to primary and secondary review. In particular:

- Triggers present on admission to hospital were not recorded as would normally be standard practice. The intention was to use the GTT to track the impact of an intervention introduced to specific areas in the hospital and so to avoid the results being affected by care outside these wards triggers existing on admission were not recorded.
- Only one harm event may be attributed to any trigger category in each individual patient i.e. if a patient had multiple falls or multiple EWS scores requiring action one harm will assigned for the trigger. This is to ensure that one patient falling repeatedly, for example, would not distort results.
- Only one harm will be attributed to a single event. It is possible for a single event to prompt two triggers e.g. an episode of hypoglycaemia is a trigger but may also lead to the administration of dextrose, a second trigger.

Below is a detailed description of the triggers and guidance on how harm will assigned.

**General care module**

**1. Early warning score**

If an early warning scoring risk assessment system is in use, then the lack of an early warning score or an early warning score requiring a response may be a precursor to an adverse event. If multiple MEWS triggers were recorded, for example if a patient deteriorated over a period of time and had multiple MEWS scores that were not appropriately responded to, this will only be counted as one harm event.

**2. Patient fall**

A fall represents a failure of care. A fall that causes no harm may be the result of medications or failure to assess risk. Any fall that causes harm regardless of cause is an adverse event by definition. Review the physician progress notes, nursing or multidisciplinary notes for evidence of over sedation, lethargy or other conditions that may have contributed to a fall.

If a patient suffered only one fall which did not lead to injury it will not be considered harm. If a second fall occurs and adequate assessment and falls prevention was not documented this will be considered a harm event. If a patient had multiple falls only one harm will be assigned. This is to ensure that results are not distorted by a single patient suffering multiple, unpreventable falls.

**3. Decubiti**

Decubitus ulcers are adverse events. Chronic decubiti are events if they occurred during a hospitalisation. Pressure ulcers present on admission will not be recorded. Reddened pressure areas will not be counted as harm.

**4. Readmission within 30 days**

Review the record to assess whether the current admission resulted in another future hospitalisation within 30 days of discharge. Details of the reason for current admission, a brief description of the treatment received and discharge planning should be recorded. The reason for readmission should also be described to allow determination of whether the readmission was due to a failure in care.

**5. Shock or cardiac arrest / crash calls**

All cardiac arrests need to be carefully reviewed as the end event of a flawed care process. However, not all crash calls are adverse events. A sudden cardiac arrhythmia with a resulting crash call may well be associated with no adverse event, but failing to rescue a patient due to lack of recognition of physiological change in signs and symptoms would be an adverse event.

**6. X-Ray or Doppler studies for emboli or Deep Vein Thrombosis (DVT)**

Development of a DVT or pulmonary embolism (PE) during a hospital stay should be considered as an adverse event. Even if all appropriate preventive measures appear to have been taken, from a patient’s perspective this is a harmful event. DVTs or PEs present on admission will not be recorded.

**7. Complication of treatment of procedure**

Evaluate the reason for the procedure. The procedure itself may be required due to an adverse event. Look for complications from any procedures or treatments. Procedure notes do not always note the complications especially if the complication occurs hours or days after the procedure note has been documented.

**8. Transfer to higher level of care**

Transfers include either within hospital, to another hospital, or to your hospital from another. Transfer to an intensive care unit or high dependency unit is a trigger that an adverse event may have occurred. Admissions to intensive care or HDU may have occurred when a patient’s clinical condition deteriorated perhaps secondary to an adverse event. When reviewing this trigger, look for the reasons for the transfer and the change in condition. For example, in the case of admission to intensive care following respiratory arrest and intubation, if the respiratory arrest was a natural progression of an exacerbation of chronic obstructive pulmonary disease (COPD), it would not be an adverse event, but if it was caused by a pulmonary embolism that developed post-operatively, or over-sedation of a patient with COPD it would be an adverse event.

**Intensive care module**

**9. Readmission to Intensive Care or High Dependency Care**

Any readmission to the ICU has a high probability of an adverse event occurring. The readmission itself may be the result of harm. Look for a relationship to a precipitating adverse event. Examples might be pulmonary oedema secondary to excess fluid administration or an aspiration.

**10. Unplanned transfer to intensive care or high dependency care**

Transfer to an intensive care unit or cardiac care unit is a trigger that an adverse event may have occurred. The admission to intensive or critical care may have occurred when a patient’s clinical condition deteriorated perhaps secondary to an adverse event. When reviewing this trigger, look for the reasons for the transfer and the change in condition. For example, in the case of admission to intensive care following respiratory arrest and intubation, if the respiratory arrest was a natural progression of an exacerbation of chronic obstructive pulmonary disease (COPD), it would not be an adverse event, but if it was caused by a pulmonary embolism that developed post-operatively, or over-sedation of a patient with COPD it would be an adverse event.

**Medication module**

**11. Vitamin K**

If Vitamin K was used as a response to a prolonged INR, review the chart for evidence of bleeding. The laboratory reports should indicate a drop in haematocrit or positive stools. Check the progress notes for evidence of excessive bruising gastrointestinal (GI) bleed, hemorrhagic stroke, large haematomas or other bleeding episodes. Record details of anticoagulation being received by the patient.

**12. Naloxone**

Naloxone is a powerful narcotic antagonist. If it has been used, over dosage of narcotics is a frequent finding.

**13. Flumazenil (Romazicon)**

Flumazenil reverses benzodiazepine drugs. Determine why the drug was used. If hypotension or marked, prolonged sedation occurred following benzodiazepine administration, an adverse event has occurred.

**14. Glucagon or 50% glucose**

The administration of glucagon or 50% dextrose indicated that the patient may have experienced an adverse event related to hypoglycaemia. The chart should be reviewed for associated use of insulin or oral hypoglycaemics with evidence of symptoms which are commonly followed by and administration of glucose (oral or intravenous). The first use of glucagons or dextrose in a non diabetic patient will not be counted as harm.

**15. Abrupt medication stop**

While some medication courses such as antibiotics are of limited duration, the cessation of several medications at once or cessation of a long term medication, such as an antihypertensive is a trigger requiring further investigation and may indicate an adverse drug reaction, drug interaction, sudden change in the patients’ condition or an unintentional omission of a drug. The cessation of long term medications for patients documented to be for palliative care or placed on the LCP will not be counted.

**Lab test module**

**Haematology**

**16. High INR (>5)**

Look for evidence of bleeding to determine if an adverse event has occurred. An elevated INR in itself is not an adverse event. Record patient anticoagulation, if a patient’s anticoagulation was not adjusted appropriately in response to an abnormal INR this would be assessed as a harm event. See also trigger 18.

**17. Transfusion**

Procedures can require intra-operative transfusion of blood products for replacement of estimated blood lost, but this has become less common with ‘bloodless surgery’. Any transfusion of packed red blood cells (RBC) or whole blood should be investigated for causation including excessive bleeding, unintentional trauma of a blood vessel, etc. Transfusion of many units within the first 24 hours of surgery, including intra-operatively and post-operatively, will commonly be related to a peri-operative adverse event. Exceptions would be where excessive blood loss occurred pre-operatively. Fresh frozen plasma and platelets can reflect system problems that include failure to plan changes in anticoagulants prior to surgery and the necessity to reverse quickly in order to do the surgery.

**18. Abrupt drop in Hb or Hct (>25%)**

Any drop of 25% or greater in Hg grams or Heamatocrit (Hct) requires an explanation. All bleeding associated events might commonly see this as a trigger. Smaller “drops” obviously can also be associated with adverse events, but the question as to whether harm occurred needs to be subjectively answered. Anticoagulant use is frequently observed to be associated with this particular trigger.

**Biochemistry**

**19. Rising urea or creatinine (>2x baseline)**

Review laboratory records for rising levels of either BUN or serum creatinine. If a change of two times greater than baseline levels is found, review medication administration records for medications known to cause renal toxicity. Review physician progress notes and the history and physical other causes of renal failure, such as pre-existing renal disease or diabetes that could have put the patient at greater risk for renal failure. Subjective judgment may be needed to determine whether renal failure was event induced if multiple factors are identified.

**20. Electrolyte abnormalities Na+ <120 or >160**

Electrolyte imbalance can either precede or be associated with adverse events. Not all patients with electrolyte abnormalities will be symptomatic. Review the case notes for evidence of symptoms.

**21. Hypoglycaemia (<3mmol/l)**

Not all patients will be symptomatic; if the patient is not symptomatic there is probably no adverse event. Review for associated use of insulin or oral hypoglycaemic with evidence of symptoms and commonly followed by and administration of glucose (oral or intravenous). Often the signs and symptoms description will be noted by nursing where lethargy, shakiness, etc. will be described.

**22. Raised Troponin (>1.5 ng/ml)**

An increase in troponin levels may indicate a cardiac event. Reviewers will need to use clinical judgement as to whether a cardiac event has occurred.

**23. K+ <2.5 or >6.0.**

Electrolyte imbalance can either precede or be associated with adverse events. Not all patients with electrolyte abnormalities will be symptomatic. Review the case notes for evidence of symptoms.

**Microbiology**

**24. MRSA bacteraemia**

Review for any positive MRSA bacteraemia. Any hospital acquired MRSA bacteraemia will be considered a harm.

**25. C. difficile**

If a patient is on or has been on multiple antibiotics this adverse event can be observed. A positive C. difficile assay is an adverse event.

**26. VRE**

Review for any nosocomial infections, central line infection, and surgical site infection or urinary tract infections. Any infection occurring in hospital is an adverse event. Exceptions might be the urinary tract infection from outside the hospital, or infection being treated but not contracted in hospital.

**27. Wound infection**

Review for any nosocomial infections, central line infection, and surgical site infection or urinary tract infections. Any infection occurring in hospital is an adverse event. Exceptions might be infection being treated but not contracted in hospital.

**28. Nosocomial pneumonia**

Any pneumonia diagnosed in the ICU needs to be looked at carefully. Any infection starting in hospital needs to be considered nosocomial unless clearly from outside the hospital. Readmissions could also represent a pneumonia from a previous hospitalisation, particularly if antibiotic resistant. All hospital acquired pneumonias will be considered a harm.

**29. Positive blood culture**

A positive blood culture at any time during hospitalisation must be investigated as an indicator of an adverse event. A surgical site infection, sepsis, infected lines or any other hospital-acquired infection is an adverse event.

**REFERENCES**

Griffen FA, Resar RK. The IHI Global Trigger Tool for Measuring Adverse Events (2^nd^ Edition) IHI Innovation Series white paper. Cambridge, Massachusetts: Institute of Healthcare Improvement 2009.

Landringhan, CP, Parry GJ, Bones CB, Hackbarth AD, Goldman DA, Sharek PJ. Temporal Trends in Rates of Harm resulting from Medical Care. New England Journal of Medicine, November 2010 363/22(2124-34)

NHS Institute for Innovation and Improvement, The IHI Global Trigger Tool for Measuring Adverse Events (UK Version) 2008

**ABBREVIATIONS**

**GTT**- Global Trigger Tool

**IHI**- Institute for Health Improvement

**SOP**- Standard Operating Procedure

# Figures

## Figure S1: Plot of subgroup means against variances

The dotted lines indicate negative-binomial regression models with different dispersion parameters$\theta$.

## Figure S2: Bar chart showing frequency of each type of harm for trigger categories within UFTO and DNACPR groups


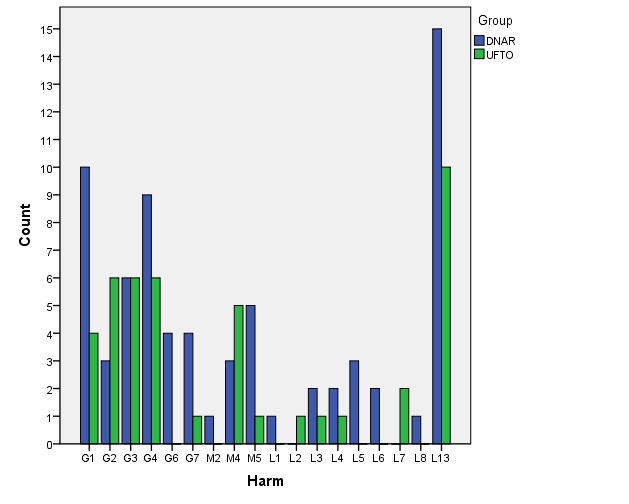


Details of triggers contained in Figure S6 Global Trigger Tool modules on page 35 of the Appendix.

## Figure S3: Bar chart showing within-group percentages of harms foreach category of severity (NCC MERP Index).


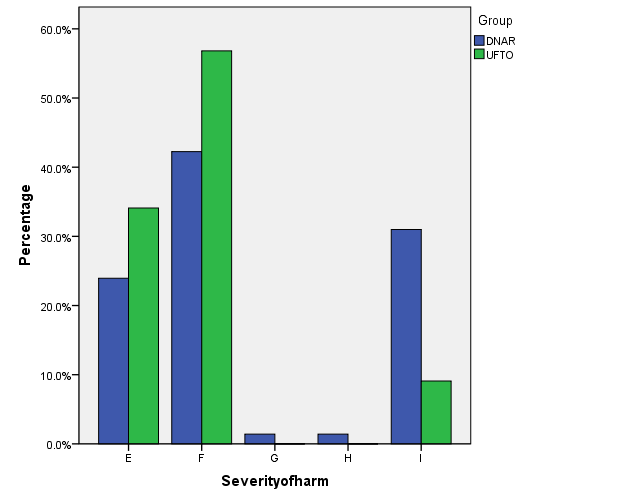


Category E: Temporary harm to the patient and required intervention

Category F: Temporary harm to the patient and required initial or prolonged hospitalisation

Category G: Permanent patient harm

Category H: Intervention required to sustain life

Category I: Patient death

## Figure S4: Bar chart showing within-group percentages of harms for each category of preventability


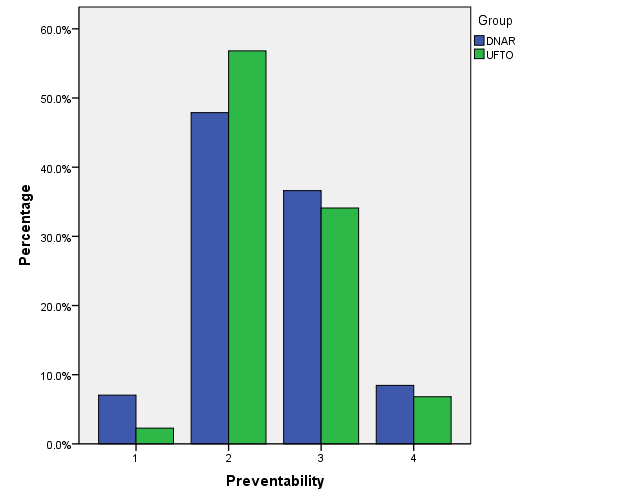


1= definitely preventable to 4= definitely not preventable

Figure S5 “Talking with your doctor about treatments: a guide for patients”


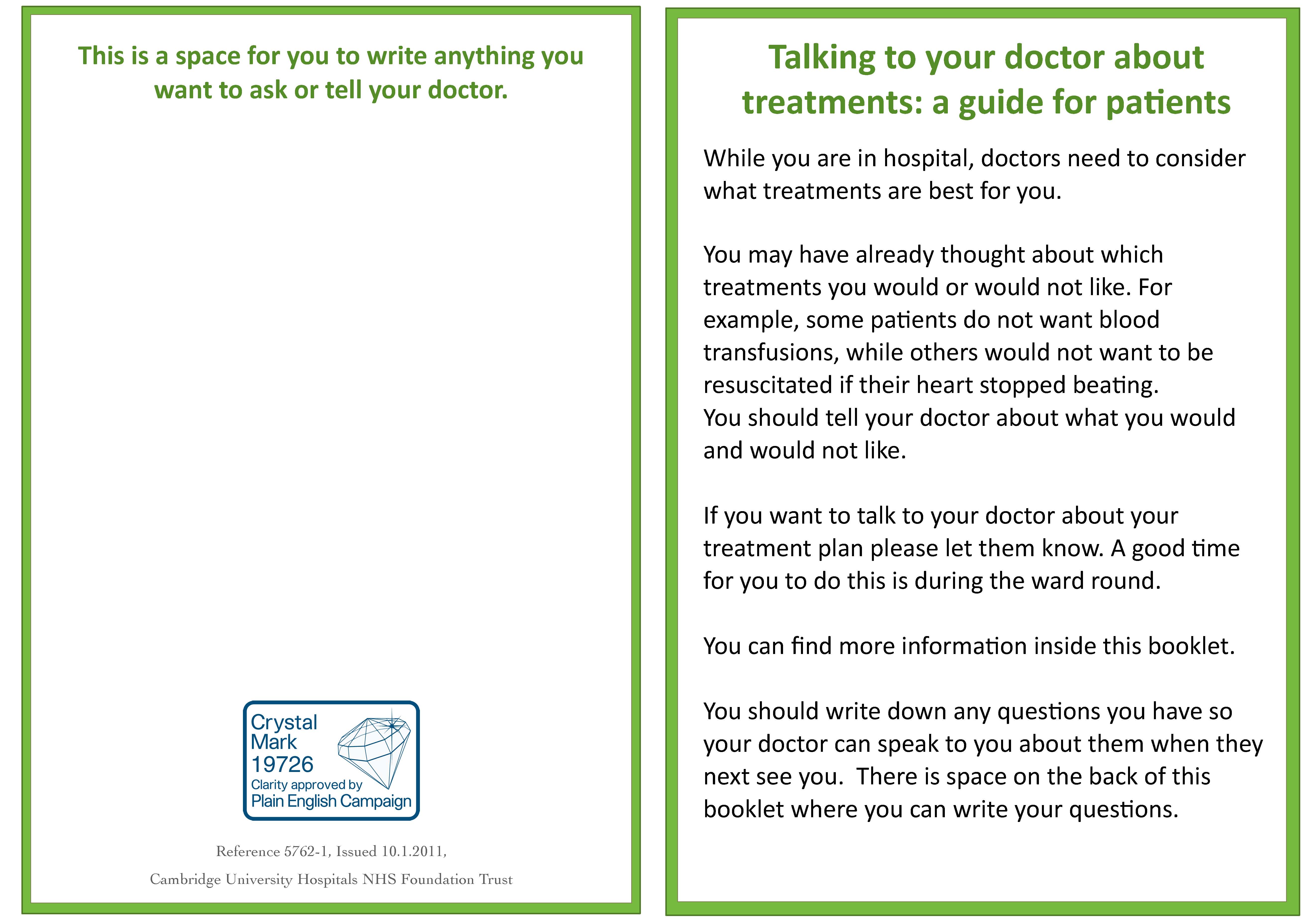

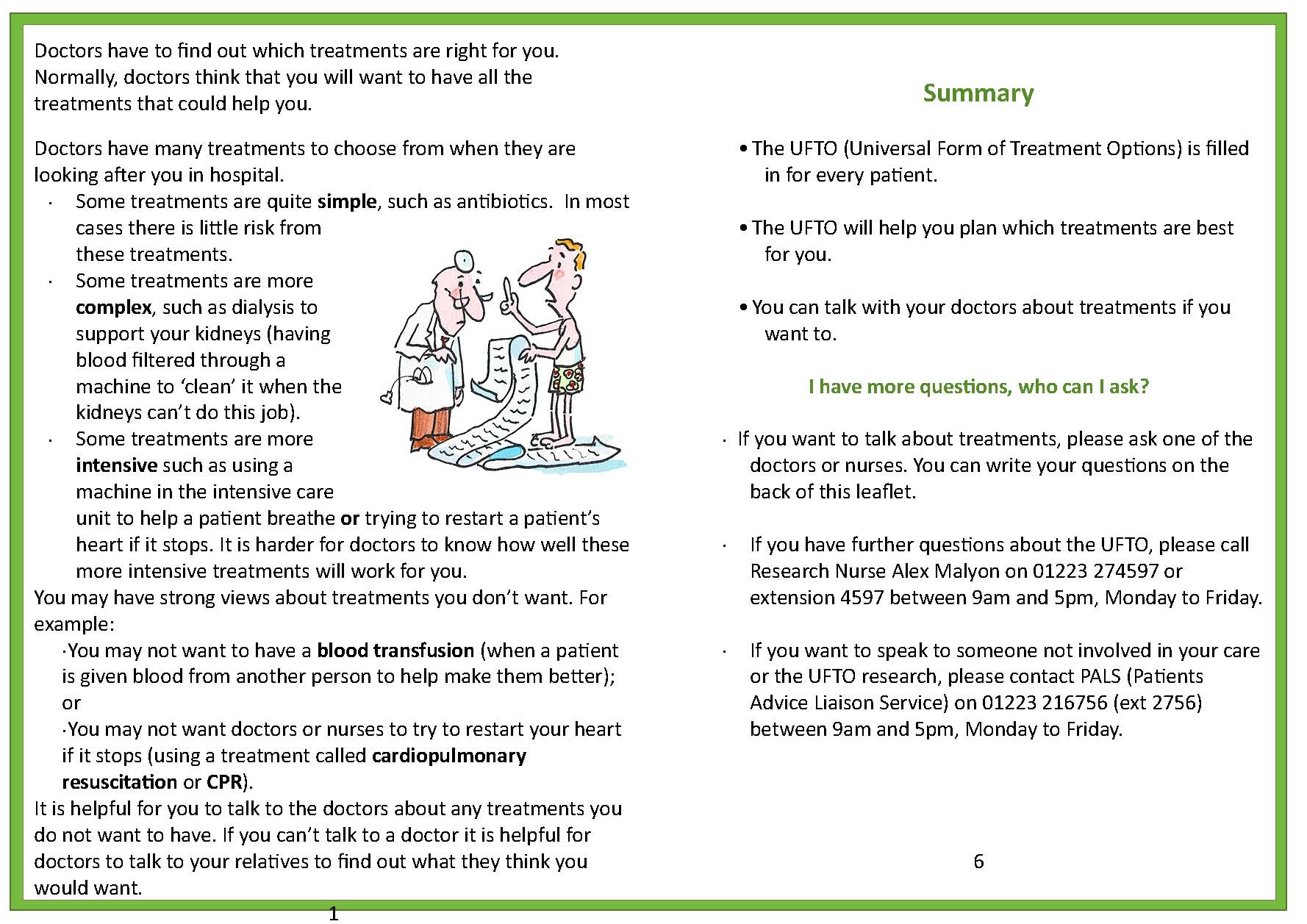

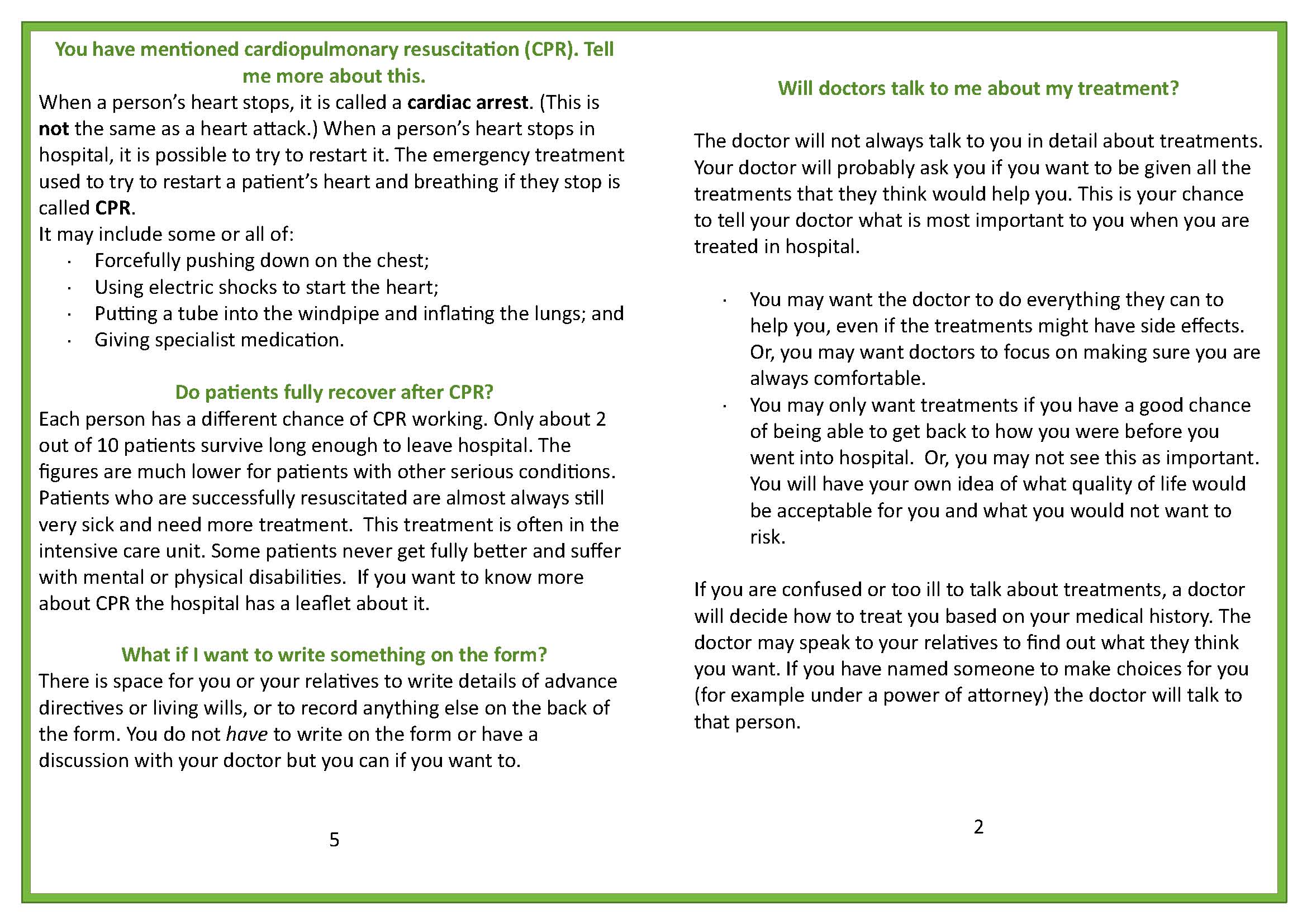

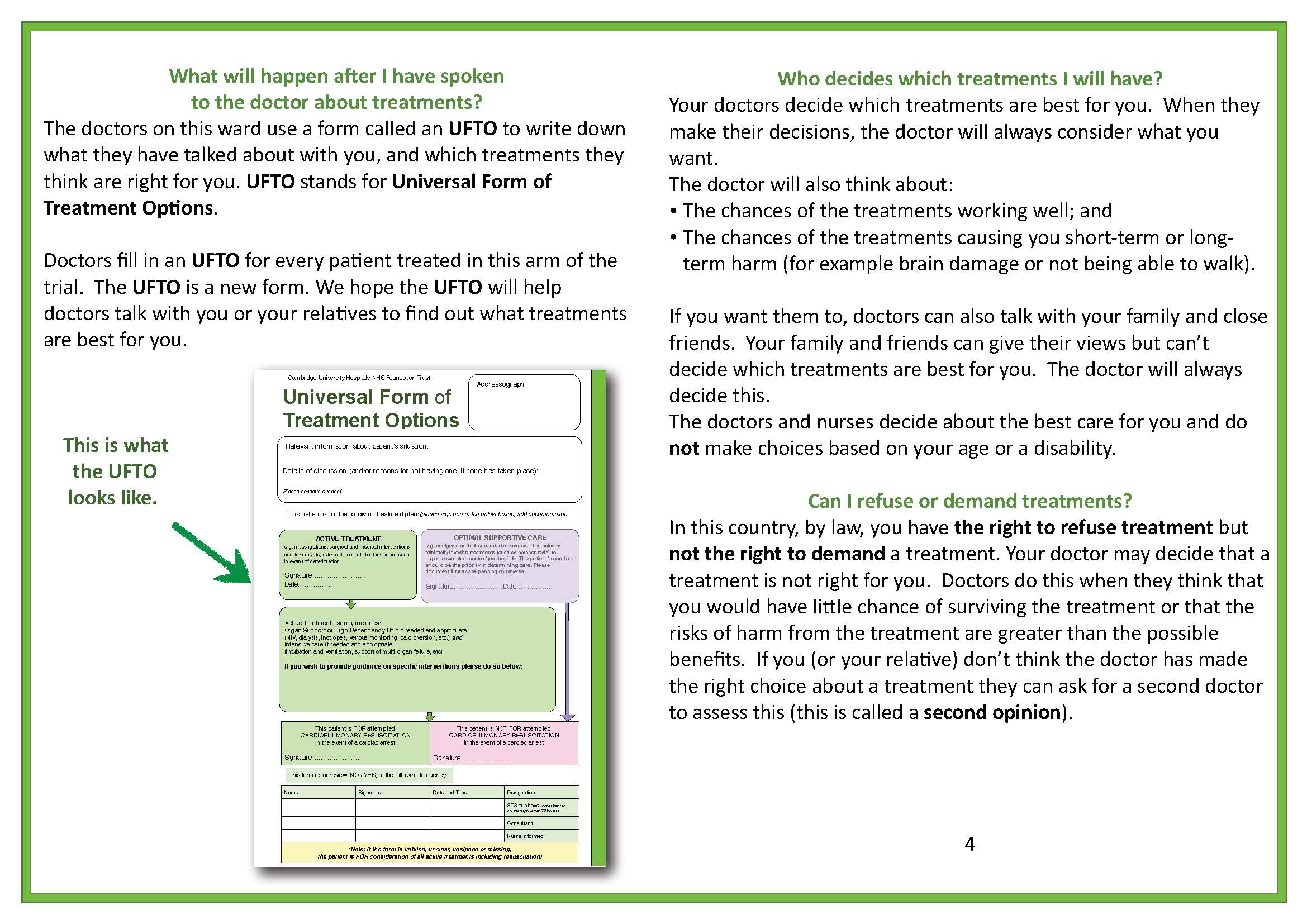


## Figure S6: the Global Trigger Tool, UK version


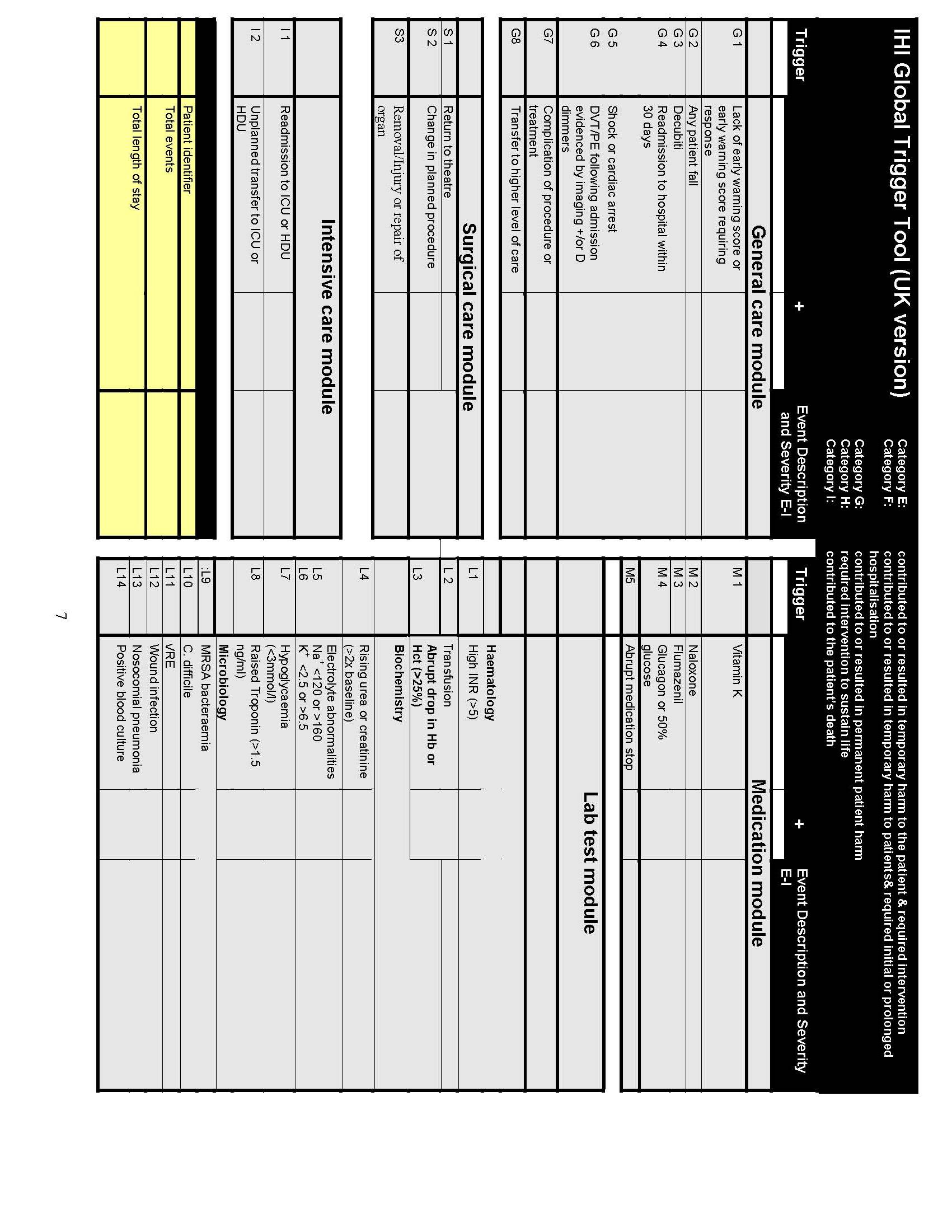

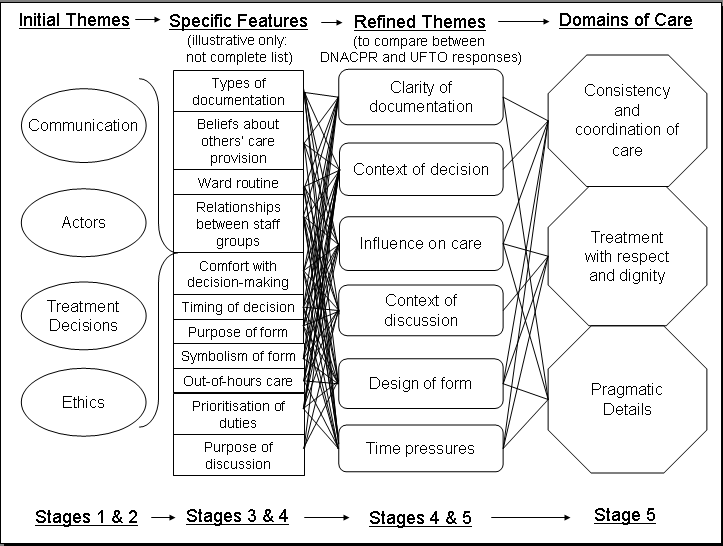


**Figure S7: Illustration of adapted Framework Analysis of interview data**

**(stages along bottom of figure correspond to Ritchie and Spencer’s (1994) 5 key stages of Framework Analysis)**


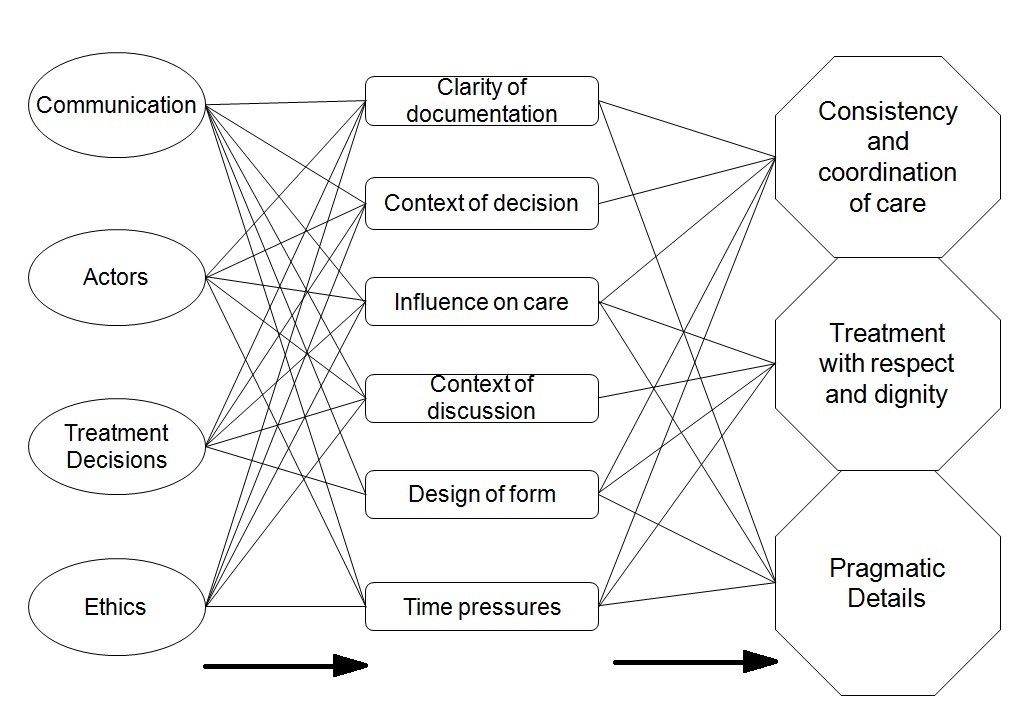


## Figure S8: Simplified illustration of how thematic framework progressed

# Tables

## Rates of harm in patients ‘Not for Resuscitation’ in the UFTO and DNACPR periods

Table S1: The average number of harms per 100 patient admissions, stratified by ward.

Approximate 95% confidence intervals are shown in brackets.

| **Ward** | **DNACPR** | **UFTO** | **Rate difference** |
| --- | --- | --- | --- |
| A | 66.7 (48.9 to 90.9) | 35.6 (24.3 to 52.3) | 31.1 (6.3 to 55.8), p=0.014 |
| B | 72.1 (50.7 to 102.5) | 40.0 (25.2 to 63.5) | 32.1 (0.7 to 63.5), p=0.045 |

Table S2: The average number of harms per 1000 patient-days, stratified by ward.

Approximate 95% confidence intervals are shown in brackets.

| **Ward** | **DNACPR** | **UFTO** | **Rate difference** |
| --- | --- | --- | --- |
| A | 40.4 (29.6 to 55.1) | 24.2 (15.2 to 38.4) | 16.2 (0.6 to 31.8), p=0.04 |
| B | 29.3 (20.6 to 41.7) | 19.0 (12.0 to 30.2) | 10.3 (-3.3 to 23.8), p=0.14 |

### Table S3: Comparison of patient characteristics between groups.

|  | **Group** | | **P-value*** |
| --- | --- | --- | --- |
|  | **DNACPR (n=103)** | **UFTO (n=118)** |  |
| Age | Mean 82.5 (SD 9.39) | Mean 82.1 (SD 9.11) | 0.77 |
| Female gender | 47 (46%) | 53 (45%) | 1.00 |
| Ward A | 60 (58%) | 73 (62%) | 0.68 |
| Length of hospital stay (days) | Median 12.0 (IQR 22.0) | Median 12.0 (IQR 16.25) | 0.86 |
| Charlson comorbidity score | Median 2.0 (IQR 3.0) | Median 2.5 (IQR 3.0) | 0.61 |
| MEWS score | Median 2.0 (IQR 3.0) | Median 2.0 (IQR 3.0) | 0.97 |

*Fisher’s exact test was used for all categorical variables and Mann-Whitney test for all continuous variables except age; for which an independent samples t-test was used.

Table S4: Regression coefficients for group (UFTO or DNACPR) expressed as per 100 patient admissions.

|  | **Explanatory variables included in model with a log-transformed offset term for hospital length of stay** | | |
| --- | --- | --- | --- |
|  | **Model 1: Group only** | **Model 2: Group and ward** | **Model 3: Group, ward, gender, Charlson comorbidity score, MEWS score and age at admission** |
| Poisson regression model | 46.5  (95% CI 8.9 to 84.1), p=0.02  AIC: 439.9 | 47.9 (95% CI 10.3 to 85.6),  p = 0.01  AIC: 440.3 | 48.8 (95% CI 10.3 to 87.2), p = 0.01  AIC: 446.4 |
| Negative binomial model | 51.7 (95% CI 12.0 to 91.5), p=0.01  AIC: 441.5 | 51.1 (95% CI 12.0 to 90.2), p=0.01  AIC: 441.6 | 51.3 (95% CI 11.6 to 91.1), p=0.01  AIC: 448.1 |

## Rates of harms for patients ‘Not for Resuscitation’ in the DNACPR and UFTO period- including palliative care patients

Table S5: The average number of harms per 100 patient admissions, stratified by ward.

Approximate 95% confidence intervals are shown in brackets.

| **Ward** | **DNACPR** | **UFTO** | **Rate difference** |
| --- | --- | --- | --- |
| A | 63.5 | 34.6 | 28.9 (5.4 to 52.4), p=0.016 |
| B | 71.1 | 33.3 | 37.8 (8.9 to 66.6), p=0.010 |

Table S6: The average number of harms per 1000 patient-days, stratified by ward.

Approximate 95% confidence intervals are shown in brackets.

| **Ward** | **DNACPR** | **UFTO** | **Rate difference** |
| --- | --- | --- | --- |
| A | 38.9 | 23.1 | 15.8 (1.1 to 30.6), p=0.04 |
| B | 29.7 | 15.8 | 13.8 (1.3 to 26.4), p=0.03 |

Table S7: Comparison of patient characteristics between groups:

|  | **Group** | | **P-value*** |
| --- | --- | --- | --- |
|  | **DNACPR (n=103)** | **UFTO (n=118)** |  |
| Age | Mean 82.3 (SD 9.62) | Mean 82.0 (SD 9.19) | 0.83 |
| Female gender | 49 (45%) | 63 (46%) | 1.00 |
| Ward A | 63 (58%) | 81 (59%) | 1.00 |
| Length of hospital stay (days) | Median 12.0 (IQR 22.0) | Median 12.0 (IQR 17.0) | 0.68 |
| Charlson comorbidity score | Median 2.5 (IQR 3.0) | Median 3.0 (IQR 3.0) | 0.33 |
| MEWS score | Median 2.0 (IQR 3.0) | Median 2.0 (IQR 3.0) | 0.95 |

*Fisher’s exact test was used for all categorical variables and Mann-Whitney test for all continuous variables except age; for which an independent samples t-test was used.

### Table S8: Regression coefficients for group (UFTO or DNACPR) expressed as per 100 patient admissions.

|  | **Explanatory variables included in model with a log-transformed offset term for hospital length of stay** | | |
| --- | --- | --- | --- |
|  | **Model 1: Group only** | **Model 2: Group and ward** | **Model 3: Group, ward, gender, Charlson comorbidity score, MEWS score and age at admission** |
| Poisson regression model | 56.3  (95% CI 19.5 to 93.0), p=0.003  AIC: 472.9 | 56.7 (95% CI 20.0 to 93.5),  p = 0.002  AIC: 472.0 | 55.7 (95% CI 18.0 to 93.4), p = 0.004  AIC: 477.0 |
| Negative binomial model | 62.5 (95% CI 23.1 to 102.0), p=0.002  AIC: 473.4 | 61.2 (95% CI 22.4 to 100.0), p=0.002  AIC: 473.3 | 59.7 (95% CI 20.2 to 99.3), p=0.003  AIC: 478.3 |

### Table S9: The frequency of each type of harm for trigger categories within UFTO and DNACPR groups

| Trigger | Frequencies of harms per group | |
| --- | --- | --- |
|  | DNACPR | UFTO |
| L13(Nosocomial pneumonia) | 15 (21%) | 10 (23%) |
| G1 (EWS requiring response) | 10 (14%) | 4 (9%) |
| G4 (Readmission witin 30 days) | 9 (13%) | 6 (14%) |
| G3 (Decubiti) | 6 (8%) | 6 (14%) |
| M5 (Abrupt medication stop) | 5 (7%) | 1 (2%) |
| G7 (Complication of treatment) | 4 (6%) | 1 (2%) |
| G6 (DVT/PE) | 4 (6%) | 0 |
| G2 (Fall) | 3 (4%) | 6 (14%) |
| M4 (Glucagon or 50% Dex) | 3 (4%) | 5 (11%) |
| L5 (Abnormal Na+) | 3 (4%) | 0 |
| L3 (>25% drop in Hb) | 2 (3%) | 1 (2%) |
| L4 (Rising Urea or creatinine) | 2 (3%) | 1 (2%) |
| L6 (Abnormal K+) | 2 (3%) | 0 |
| M2 (Naloxone administered) | 1 (1%) | 0 |
| L1 (High INR) | 1 (1%) | 0 |
| L8 (Raised Troponin) | 1 (1%) | 0 |
| L7 (Hypoglycaemia) | 0 | 2 (5%) |
| L2 (Transfusion) | 0 | 1 (2%) |
| Total harms | 71 | 44 |

### Table S10: Rating of severity of harms using NCC MERP Index severity of the harms in DNACPR and UFTO groups

|  | | Group | | Total |
| --- | --- | --- | --- | --- |
|  |  | DNACPR | UFTO |  |
| Severity* | E | 17 | 15 | 32 |
|  | F | 30 | 25 | 55 |
|  | G | 1 | 0 | 1 |
|  | H | 1 | 0 | 1 |
|  | I | 22 | 4 | 26 |
| Total | | 71 | 44 | 115 |

Category E: Temporary harm to the patient and required intervention

Category F: Temporary harm to the patient and required initial or prolonged hospitalisation

Category G: Permanent patient harm

Category H: Intervention required to sustain life

Category I: Patient death

### Table S11: severity of harms collapsed in preparation for a Chi-squared test for trend (with all expected cell values more than 4)

|  | | Group | | Total |
| --- | --- | --- | --- | --- |
|  |  | DNACPR | UFTO |  |
| Severity* | E | 17 | 15 | 32 |
|  | F | 30 | 25 | 55 |
|  | G, H, I | 24 | 4 | 28 |
| Total | | 71 | 44 | 115 |

### Table S12: The distribution of preventability of harms in each group.

| Preventability* | | Group | | Total |
| --- | --- | --- | --- | --- |
|  |  | DNACPR | UFTO |  |
|  | 1 | 5 | 1 | 6 |
|  | 2 | 34 | 25 | 59 |
|  | 3 | 26 | 15 | 41 |
|  | 4 | 6 | 3 | 9 |
| Total | | 71 | 44 | 115 |

*1= definitely not preventable 4= definitely preventable

### Table S13: preventability of harms collapsed in preparation for a Fisher’s Exact test

|  | | Group | | Total |
| --- | --- | --- | --- | --- |
|  |  | DNACPR | UFTO |  |
| Preventability | 1-2 | 39 | 26 | 65 |
|  | 3-4 | 32 | 18 | 50 |
| Total | | 71 | 44 | 115 |

## Contemporaneous controls- patients not for resuscitation on wards not participating in study

### Table S14: Comparison of patient characteristics between groups for patients on non study wards during the DNACPR period (May-July 2010) and UFTO period (Dec 2010-Jan 2011)

|  | **Period** | | **P-value*** |
| --- | --- | --- | --- |
|  | **DNACPR period (n=25)** | **UFTO period (n=25)** |  |
| Age | Median 85.5 (IQR 9.1) | Median 83.4 (IQR 10.9) | 0.38 |
| Female gender | 17 (68%) | 16 (64%) | 1.00 |
| Control wards | 19 (76%) | 17 (68%) | 0.75 |
| Length of hospital stay (days) | Median 21.0 (IQR 25.5) | Median 18.0 (IQR 24.0) | 0.25 |
| Charlson comorbidity score | Median 2.0 (IQR 2.0) | Median 3.0 (IQR 3.5) | 0.04 |
| MEWS score | Median 2.0 (IQR 2.0) | Median 2.0 (IQR 3.0) | 0.68 |

Fisher’s exact test was used for all categorical variables and Mann-Whitney test for all continuous variables.

### Table S15a: Comparison between characteristics of patients not for resuscitation on study wards and on control wards during the DNACPR period.

|  | **Group** | | **P-value*** |
| --- | --- | --- | --- |
|  | **Study (n=103)** | **Controls (n=25)** |  |
| Age | Mean 82.5 (SD 9.4) | Mean 84.0 (SD 8.4) | 0.47 |
| Female gender | 47 (46%) | 17 (68%) | 0.07 |
| Length of hospital stay (days) | Median 12.0 (IQR 22.0) | Median 21.0 (IQR 25.5) | 0.02 |
| Charlson comorbidity score | Median 2.0 (IQR 3.0) | Median 2.0 (IQR 2.0) | 0.16 |
| MEWS score | Median 2.0 (IQR 3.0) | Median 2.0 (IQR 2.0) | 0.85 |

*Fisher’s exact test was used for the categorical gender variable and Mann-Whitney test for all continuous variables except age; for which an independent samples t-test was used.

***Table S15b: Comparison between characteristics of patients not for resuscitation on study wards and on control wards during the UFTO period.***

|  | **Group** | | **P-value*** |
| --- | --- | --- | --- |
|  | **Study (n=118)** | **Controls (n=25)** |  |
| Age | Mean 82.1 (SD 9.1) | Mean 82.0 (SD 9.6) | 0.97 |
| Female gender | 53 (45%) | 16 (64%) | 0.12 |
| Length of hospital stay (days) | Median 12.0 (IQR 16.25) | Median 18.0 (IQR 24.0) | 0.31 |
| Charlson comorbidity score | Median 2.5 (IQR 3.0) | Median 3.0 (IQR 3.5) | 0.22 |
| MEWS score | Median 2.0 (IQR 3.0) | Median 2.0 (IQR 3.0) | 0.72 |

*Fisher’s exact test was used for the categorical gender variable and Mann-Whitney test for all continuous variables except age; for which an independent samples t-test was used.

### Table S16: Regression coefficients for group (UFTO or DNACPR) on control wards expressed as per 100 patient admissions.

|  | **Explanatory variables included in model with a log-transformed offset term for hospital length of stay** | | |
| --- | --- | --- | --- |
|  | **Model 1: Group only** |  | **Model 3: Group, ward, gender, Charlson comorbidity score, MEWS score and age at admission** |
| Poisson regression model | 57.8 (95% CI -14.5 to 130.0), p=0.12  AIC: 113.7 |  | 53.4 (95% CI -22.1 to 128.9),  p = 0.17  AIC: 111.7 |
| Negative binomial model | 45.0 (95% CI -55.0 to 144.9), p=0.38  AIC: 108.4 |  | 52.8 (95% CI -36.2 to 141.8), p=0.25  AIC: 112.8 |

### Table S17: Comparison of patient characteristics between groups (UFTO or DNACPR periods) for patients remaining for resuscitation on study wards.

|  | **Group** | | **P-value*** |
| --- | --- | --- | --- |
|  | **DNACPR (n=60)** | **UFTO (n=58)** |  |
| Age | Median 75.5 (IQR 18.2) | Median 79.1 (IQR 18.8) | 0.46 |
| Female gender | 29 (48%) | 26 (45%) | 0.72 |
| Ward A | 32 (53%) | 34 (59%) | 0.58 |
| Length of hospital stay (days) | Median 5.5 (IQR 8.0) | Median 6.0 (IQR 8.5) | 0.47 |
| Charlson comorbidity score | Median 1.5 (IQR 1.0) | Median 2.0 (IQR 2.0) | 0.64 |
| MEWS score§ | Median 2.0 (IQR 2.0) | Median 2.0 (IQR 3.0) | 0.41 |

*Fisher’s exact test was used for all categorical variables and Mann-Whitney test for all continuous variables.

§ Missing MEWS score for one DNACPR patient.

### Table S18: Exclusions from dataset on study wards during DNACPR and UFTO periods.

|  | DNACPR period | UFTO period |
| --- | --- | --- |
| Total included admissions | 513 | 520 |
| Missing notes | 1 | 2 |
| Palliative/OSC | 5 | 21 |
| Excl <24 hrs | 9 | 13 |
| Excl <18yrs | 2 | 3 |
| Other | 1 | 3 |
| Total Excluded | 18 | 42 |
| Total excluded apart from Palliative care/OSC | 13 | 21 |

## Mortality Tables

### Table S19: 30 day mortality in those patient not for resuscitation

|  | DNAR period | UFTO period |  |
| --- | --- | --- | --- |
| Ward A | 16 | 14 |  |
| Ward B | 12 | 13 |  |
| Other ward | 8 | 4 |  |
| Out of hospital | 10 | 5 |  |
| Total deaths 30 day | 45 | 35 |  |

### Table S20:30 day mortality in palliative care patients

|  | DNAR period (5 total) | UFTO period (25 total) |  |
| --- | --- | --- | --- |
| Ward A | 1 | 3 |  |
| Ward B | 1 | 7 |  |
| elsewhere | 2 | 6 |  |
| Total deaths 30 day | 4 | 16 |  |

### Table S21:30 day mortality for other excluded patients

|  | DNAR period | UFTO period |
| --- | --- | --- |
| Admission <24hrs Ward A | 2 | 1 |
| Ward B | 0 | 1 |
| Other ward | 0 | 1 |
| <18years of age | 0 | 0 |
| Other | 0 | 2 |
| Total deaths 30day | 2 | 5 |

### Table S22: 30 day mortality in those patients for resuscitation on the study wards

|  | DNAR period | UFTO |
| --- | --- | --- |
| Other ward | 3 | 7 |
| Non WSH | 4 | 5 |
| Other | 0 | 3 |
| Total death 30 days | 7 | 15 |

### Table S23: Total 30 day mortality

| DNAR | UFTO |
| --- | --- |
| 58/530 (10.9%) | 71/560 (12.6%) |
|  |  |

## Qualitative Tables

### Table S24: Breakdown of Interview Participants by Clinical Grade

| **Profession** | **Clinical Grade** | **DNACPR** | **UFTO** |
| --- | --- | --- | --- |
| **Doctor** | **Consultant** | 5 | 5 |
|  | **ST3 or above** | 2 | 3 |
|  | **ST1 - ST2** | 6 | 3 |
| **Nurse** | **Ward Manager** | 2 | 2 |
|  | **Sister** | 4 | 7  (3 individual interviews and 1 group interview with 4 participants) |
|  | **Staff Nurse** | 7 | 4 |

### Table S25: Identifying a thematic framework: Level 1 and 2 coding for whole data set

| **Level 1 code** | **Level 2 code** |
| --- | --- |
| UFTO | Communication |
|  | Actors |
|  | Treatment Decisions |
|  | Ethics |
| DNACPR | Communication |
|  | Actors |
|  | Treatment Decisions |
|  | Ethics |
| General | Communication |
|  | Actors |
|  | Treatment Decisions |
|  | Ethics |

### Table S26: Indexing: example of full descriptive coding labels under level 1 code 'UFTO'

| **Level 1** | **Level 2** | **Level 3** | **Level 4** | **Level 5** | **Level 6** |
| --- | --- | --- | --- | --- | --- |
| UFTO | Communication | Nursing Handover | treatment plan |  |  |
|  |  |  | Other |  |  |
|  |  | in notes | electronic record |  |  |
|  |  |  | paper notes |  |  |
|  |  | On ward |  |  |  |
|  |  | Effect on relationship |  |  |  |
|  |  | Discussions | Doctors and Patients |  |  |
|  |  |  | Doctors and relatives |  |  |
|  |  |  | Doctors and Nurses | Making decisions |  |
|  |  |  |  | Out-of-hours |  |
|  |  |  | Nurses and Patients |  |  |
|  |  |  | Nurses and relatives |  |  |
|  |  |  | Senior and Junior Drs |  |  |
|  |  |  | ward drs and specialists |  |  |
|  |  |  | comfort with topic |  |  |
|  |  |  | Purpose |  |  |
|  |  |  | Understanding |  |  |
|  |  |  | information exchanged |  |  |
|  |  |  | nurse and nurse |  |  |
|  |  | Other |  |  |  |
|  | Actors | Clinicians | Doctors | Junior Doctors |  |
|  |  |  |  | Senior Doctors | Registrars |
|  |  |  |  |  | consultants |
|  |  |  |  | Out-of-Hours Doctors |  |
|  |  |  |  | Specialist Doctors |  |
|  |  |  |  | Personal opinions | positives |
|  |  |  |  |  | negatives |
|  |  |  | Nurses | Sisters |  |
|  |  |  |  | Staff nurses |  |
|  |  |  |  | Nurse specialists |  |
|  |  |  |  | Personal opinions | positives |
|  |  |  |  |  | negatives |
|  |  | Lay people | Patients |  |  |
|  |  |  | Relatives |  |  |
|  | Treatment Decisions | Clarity |  |  |  |
|  |  | active treatment | Referrals |  |  |
|  |  |  | Investigations |  |  |
|  |  |  | Escalation |  |  |
|  |  | supportive care | LCP |  |  |
|  |  | Out-of-Hours |  |  |  |
|  |  | Advance Care Planning |  |  |  |
|  |  | End-of-Life care |  |  |  |
|  | Ethics | 'duty' |  |  |  |
|  |  | 'dilemma' |  |  |  |
|  |  | 'best interests' |  |  |  |
|  |  | 'problem' | DNACPR 'problem' | Challenges DNACPR | negatives DNACPR |
|  |  |  |  |  | improvements to DNACPR suggested |
|  |  |  |  | positives DNACPR | affirmations DNACPR |
|  |  |  |  |  | How DNACPR has improved |
|  |  |  |  | DNACPR no effect |  |
|  |  |  | UFTO 'problem' | UFTO challenges | Details of UFTO challenges |
|  |  |  |  |  | negative feelings about the UFTO |
|  |  |  |  |  | suggestions for improvement |
|  |  |  |  | UFTO – no change |  |
|  |  |  |  | improvement with UFTO | Details of improvement |
|  |  |  |  |  | affirmation of the UFTO |

From indexing the data with initial descriptive codes, we identified specific features which were relevant across these coded ‘chunks’ of data. For example, ‘timing of decisions’ was a specific feature which we identified as relevant across treatment decision-making and communications, for DNACPR and UFTO responses.

### Table S27: Example of charting: the theme of ‘Timing’

| Theme ↓ | Case → | 0002 (consultant) | 0017 (nurse) | 0032 (registrar) |
| --- | --- | --- | --- | --- |
| Timing of decision –DNACPR  (DNACPR; Treatment decisions; timing) | | “Because of the nature of the system, there’s often forms that are already signed prior to my ward rounds so often I come in to see patients who have been under a different clinician, it’s usually a geriatric consultant who’s then handed over the patient to the respiratory team because the patient’s gone to the respiratory ward so I may not necessarily have been party to that discussion of initial decision making process”  “circumstances might have changed within the course of the admission for... You know, to result in a change in approach to management... And sometimes the circumstances of the patient themselves, the way they respond to initial treatment might suggest that you need to change the decision”  “we’ve adapted ourselves and our practice to be able to make very quick decisions because it’s the nature of the work that we do”  “I think definitely it’s worth having that discussion and thought process in the clear light of day rather than having to make those decisions suddenly and via a junior person instead because it’s better to have guidance from more senior clinicians because you’re going to be able to maybe make judgements than junior staff.”  “it’s often in the context of a very sick patient that you’re talking to a relative anyway because often you don’t end up having the opportunities to speak to people when patients aren’t that sick because, you know, they won’t necessarily be there.”  “they already had the decisions made because they were transferred to my ward after they had those decisions made so it wouldn’t have been relevant for me to readdress it.” | “Ward rounds especially on a Tuesday I would say when we’ve had the new patients brought in that the Consultants haven’t seen until the Tuesday and then there’s normally quite a few DNARs put it”  “out of hours it’s a bit more, I think the ones that are done out of hours are a bit more rushed and a bit, there’s not as much discussion, it’s just, “right, we now need to put this patient”, because they’ve got to the stage where they need to be on it, whereas at least on a ward round I think they’re a bit more thought through, more time is taken out and it’s, “right, what have they got as their personal history, what is the likely outcome”, yeah, there’s a lot more factors taken in”  [about the effect of filling in DNACPR forms earlier] “would you start putting people on DNARs quicker when actually you kind of think actually they would have come through it better? I wouldn’t want any patient put on a DNAR if actually you kind of think they could have come back from that with a good chance... some people will come in very unwell but then will improve, yet that DNAR will still be in place unless someone says, ‘oh, can we recheck that?’”  “I mean quite often we have a few who the doctors might be considering an LCP and then suddenly it’s you need a decision made now because they have arrested”  “with some of our regular ones who keep coming in, who you know for a fact were DNAR last time and you do kind of say, “we’ve got this patient back in, do you want them back on it?”, and the doctor will very quickly sign it, but there’s some patients who you know, they had a DNAR a few years back but they were in a lot worse condition and they improved, so I think it’s not just have they been on one before but it’s how they’ve come in this time” | “when somebody’s unwell and it triggers in your mind that you have to make plans should a patient deteriorate. Other things would be patients with terminal diseases, so terminal malignancies, end-stage heart failure, COPD, those kind of things. But it isn’t something you would routinely do on everybody”  “if people are unwell the culture in medicine is that these DNACPR decisions are not made until they have to be. Or they might be thought about but nobody wants to take the responsibility for filling them in or doing them or anything else. Which, you know, is all well and good, you do that when you’re on take, you go home, you don’t have to worry about it – it’s somebody else’s responsibility”  “with DNACPR a lot of the times you’re kind of, you’re filling those in when people are very unwell, and it’s kind of obvious that they’re not going to get better, and so it’s fairly clear-cut”  “people generally only make DNACPR decisions and things when people deteriorate”  “‘yeh, he should never have been for CPR but he was stable and going to go home so we just didn’t fill in a form.’ That’s the problem with the old system, you know, that this chap was put through whatever, a couple of minutes of CPR, before the crash team got there , that was completely inappropriate.... the team on the ward hadn’t looked to do these things. Whether they just didn’t want to, whether it was more work for them or, like I said, they thought he was fairly stable and going home and was just having social input, you know, these patients are high risk of having anything: having a heart attacks, PEs, anything at all and things surprise you: people go off quite quickly. So, yes, he should have had a not for resus form ” |

From ‘charting’ these specific features across cases we identified themes which encompassed the relationships between them, and these became our refined themes. We used the refined themes as a basis for comparison between responses about the DNACPR form and about the UFTO. The findings of this comparison are summarised in Table 4 below, grouped together under three overarching ‘Domains of Care’ which embody the key features of our findings.

### Table S28 Stage 5 - Mapping and Interpretation: Key Themes emergent from Interview data

| **Domains of Care** | **DNACPR** | **UFTO** |
| --- | --- | --- |
| Interdisciplinary communication, clarity and consistency | Unequivocal, ‘STOP’ sign | Sense of direction/forward planning |
|  | Arbitrary, ad hoc, only at crisis point | Systematic |
|  | Marking out, ‘special case’ | Habitual, universal, routinised |
|  | Unofficial triage | General clinical summary |
|  | Insidious | Open |
| Patient dignity and respect | Potentially negative associations for patients/relatives | Normalising for patients/ relatives |
|  | Negative associations for clinicians | Normalising for clinicians |
|  | Precipitates evaluations of futility | Encourages evaluations of appropriate actions |
|  | Clinical discomfort with decision | Clinical comfort with decision |
|  | Stigma of form discourages conversations with patients and relatives | Makes clinicians more comfortable in their discussions with patients and relatives |
| Pragmatic details | Recognisable in an emergency | Recognisable in an emergency |
|  | Straightforward to complete – not demanding on time | Straightforward to complete – takes a little time but saves more time later on |
|  | Permanent record of a single clinical decision | Permanent record of a range of clinical decisions |
